# Supplementary material for: Modulating CO2 electroreduction pathways through controlled ionomer arrangement on catalyst surfaces via solvent dispersion
Source: Innovation (Camb). 2025 Mar 18;6(6):100882. doi: 10.1016/j.xinn.2025.100882 (PMC12169282; doi:10.1016/j.xinn.2025.100882)
Supplement: Document S1. Figures S1–S31 and Table S1 [file mmc1.pdf]

The Innovation, Volume *6*

Supplemental Information

Modulating CO_2 electroreduction pathways through controlled ionomer arrangement on catalyst surfaces via solvent dispersion

Yaoyu Yin, Zhongnan Ling, Shiqiang Liu, Jiapeng Jiao, Meng Zhou, Pei Zhang, Xing Tong, Yueqian Fan, Jiahao Yang, Huanyan Liu, Xueqing Xing, Jianling Zhang, Yi Xu, Hongyan Liang, Xinchen Kang, and Buxing Han

Supplementary Figures

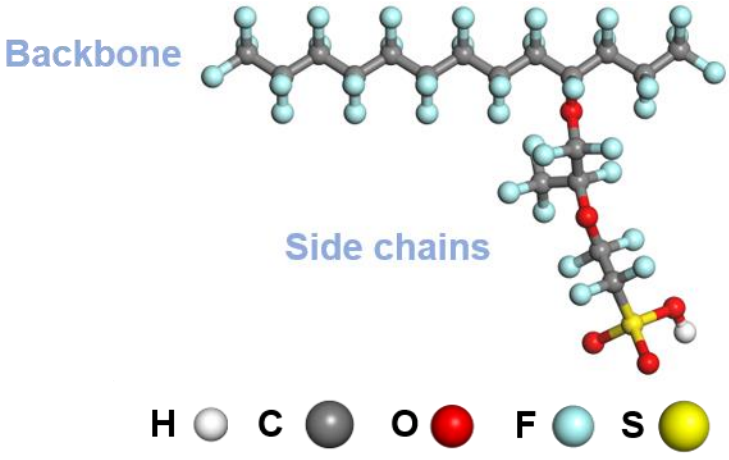

Figure S1. The structure of Nafion monomer..

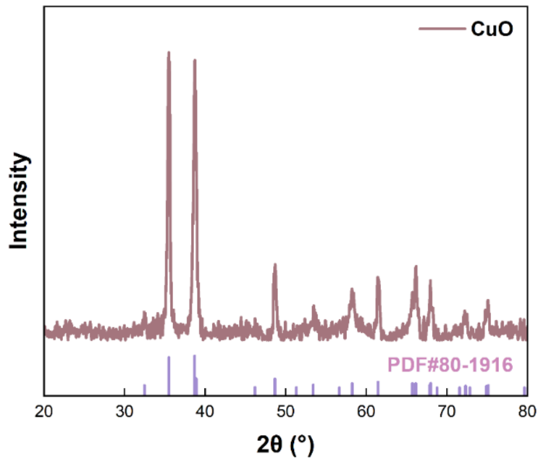

Figure S2. XRD pattern of CuO nanorods.

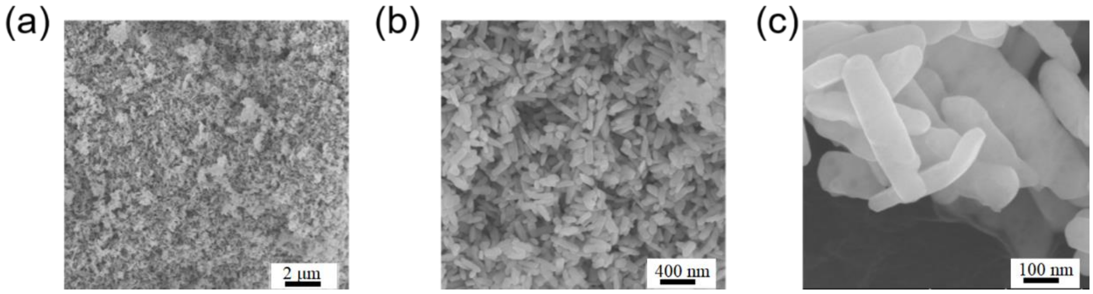

Figure S3. SEM images of CuO nanorods.

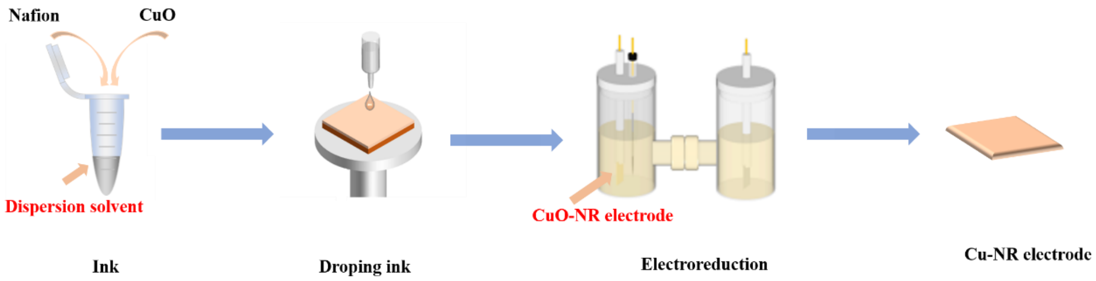

Figure S4. Schematic diagram for the preparation of Cu-NR electrodes.

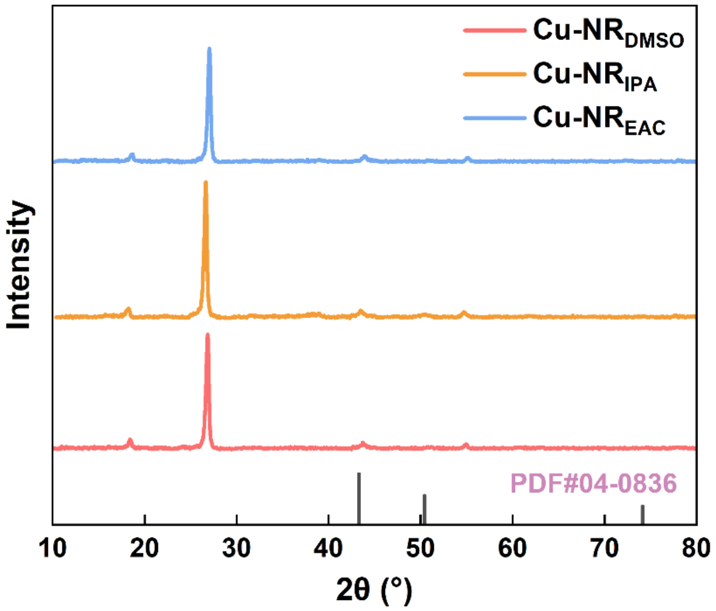

Figure S5. XRD pattern of Cu-NR_{DMSO} (a), Cu-NR_{IPA} (b) and Cu-NR_{EAC} (c).

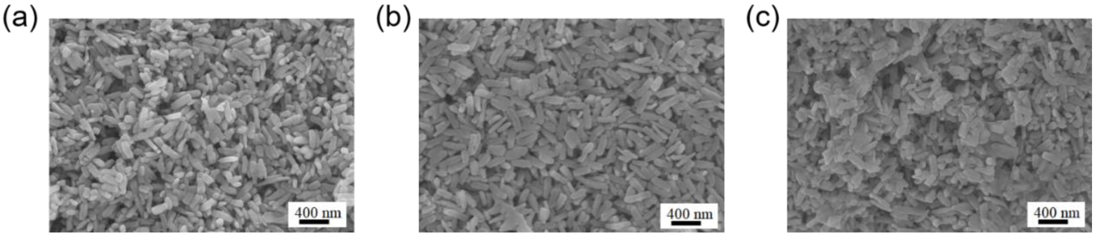

Figure S6. SEM images of Cu-NR$_{DMSO}$ (a), Cu-NR$_{IPA}$ (b) and Cu-NR$_{EAC}$ (c).

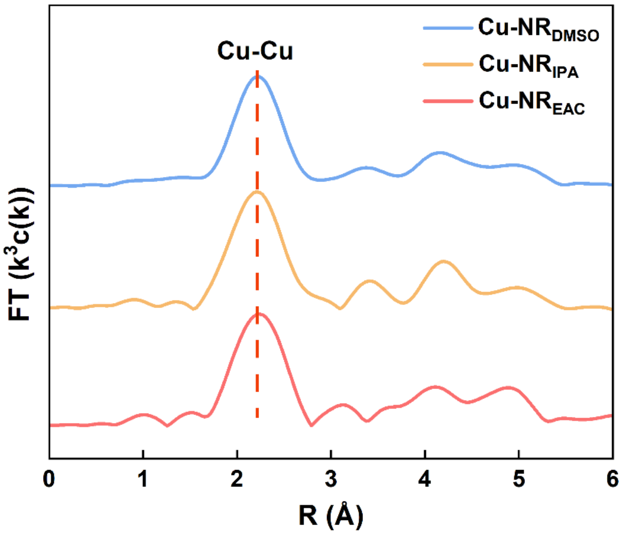

Figure S7. The Cu K-edge XANES spectra of different Cu electrodes.

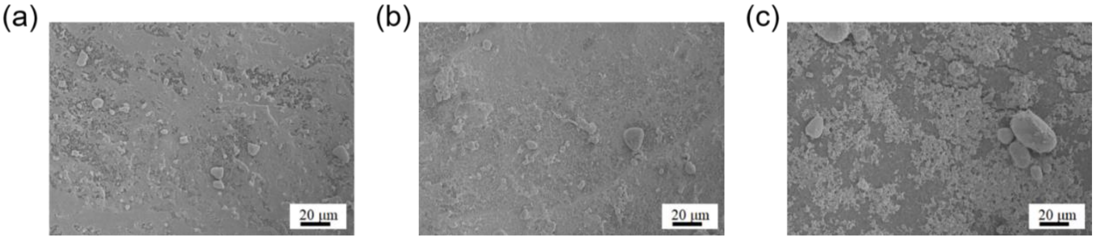

Figure S8. SEM images of Cu-NR$_{DMSO}$ (a–b), Cu-NR$_{IPA}$ (c–d) and Cu-NR$_{EAC}$ (e–f).

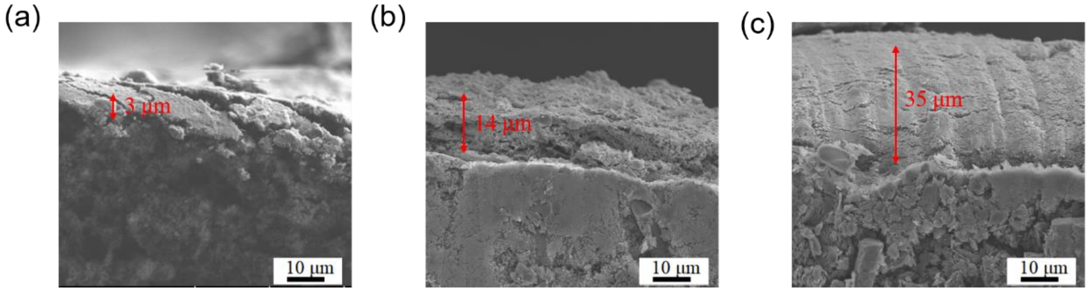

Figure S9. SEM images from side view of Cu-NR$_{DMSO}$ (a), Cu-NR$_{IPA}$ (b) and Cu-NR$_{EAC}$ (c).

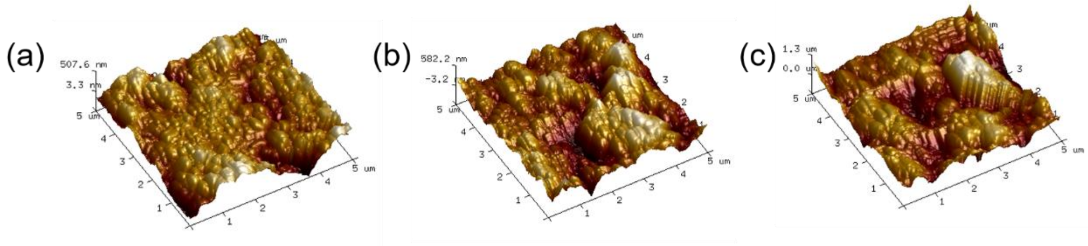

Figure S10. 3D AFM images of Cu-NR$_{DMSO}$ (a), Cu-NR$_{IPA}$ (b) and Cu-NR$_{EAC}$ (c).

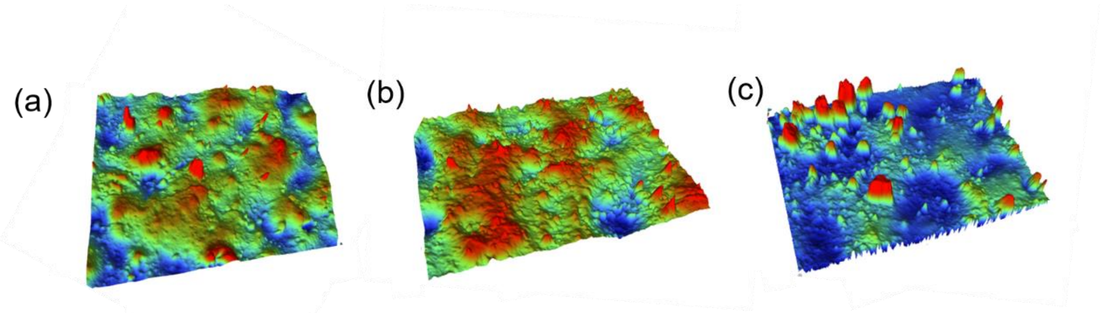

Figure S11. 3D Profiling images of Cu-NR$_{DMSO}$ (a), Cu-NR$_{IPA}$ (b) and Cu-NR$_{EAC}$ (c).

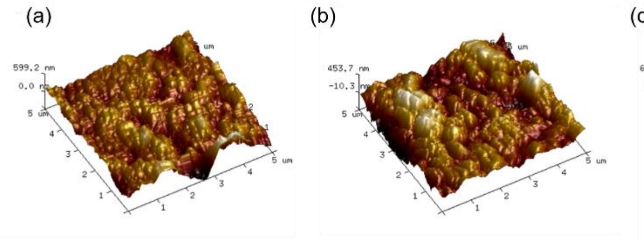

Figure S12. 3D AFM images of CuO-NR$_{DMSO}$ (a), CuO-NR$_{IPA}$ (b) and CuO-NR$_{EAC}$ (c).

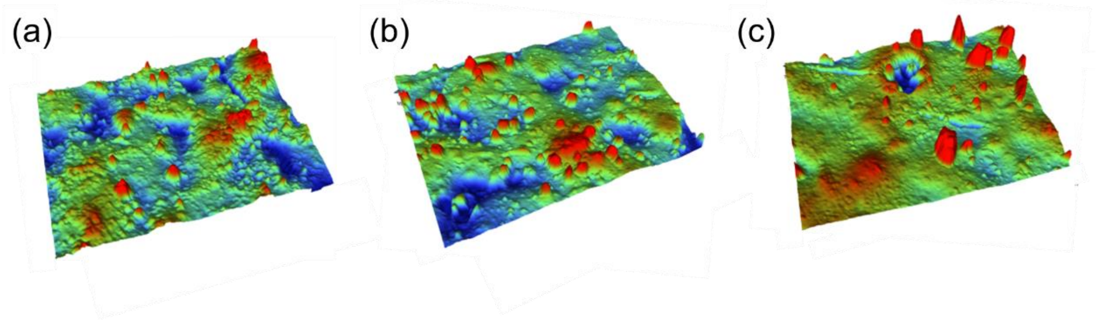

Figure S13. 3D Profiling images of CuO-NR$_{DMSO}$ (a), CuO-NR$_{IPA}$ (b) and CuO-NR$_{EAC}$ (c).

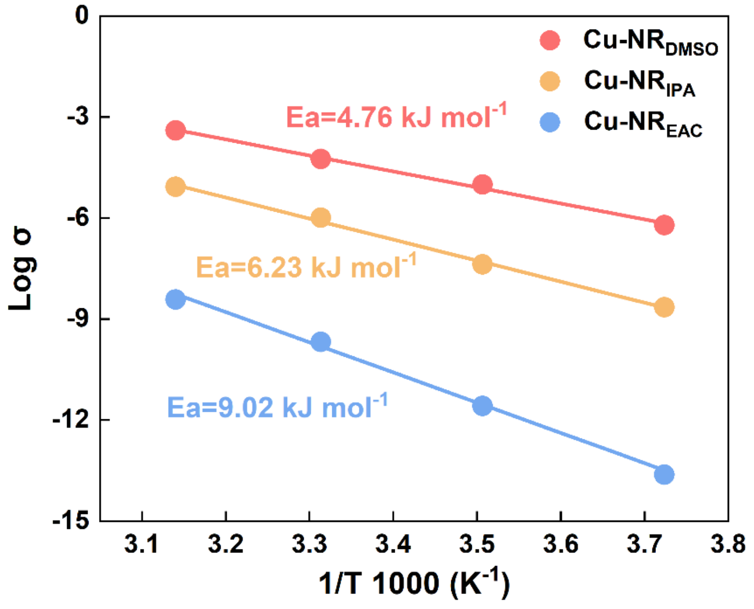

Figure S14. Relationship between conductivity and temperature.

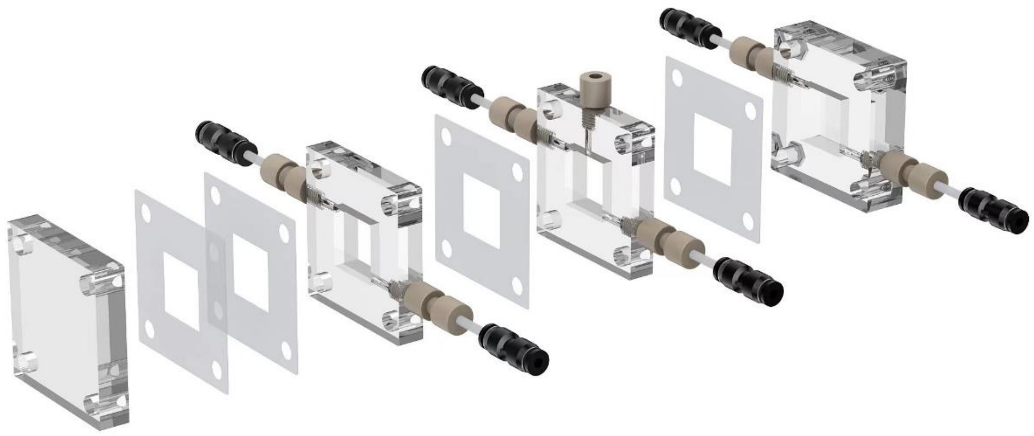

Figure S15. Schematic diagram of the flow cell used for CO_2RR.

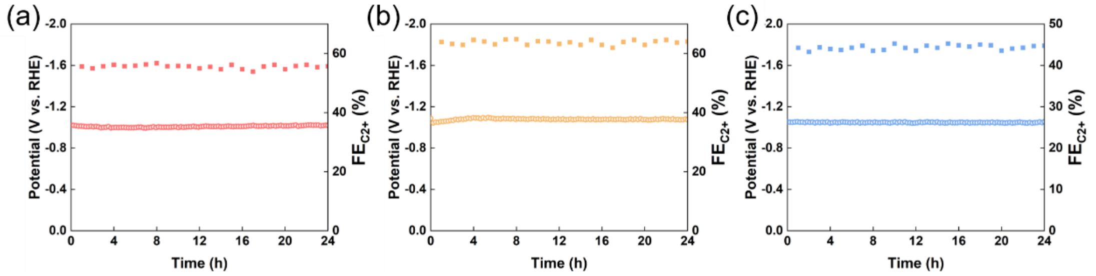

Figure S16. Plot of potential (hollow circle) and FE_{C2+} (solid circle) vs. time over Cu-NR_{DMSO} (a), Cu-NR_{IPA} (b) and Cu-NR_{EAC} (c) at 800 mA cm^{-2}.

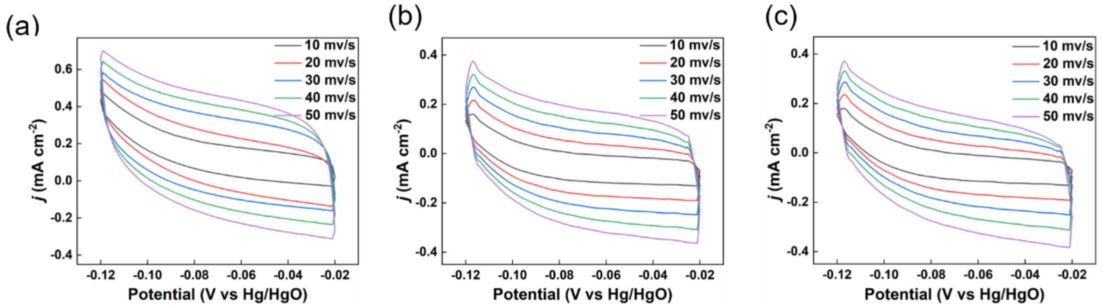

Figure S17. CV scans at the non-Faradaic region with different scan rates over Cu-NR_{DMSO} (a), Cu-NR_{IPA} (b) and Cu-NR_{EAC} (c).

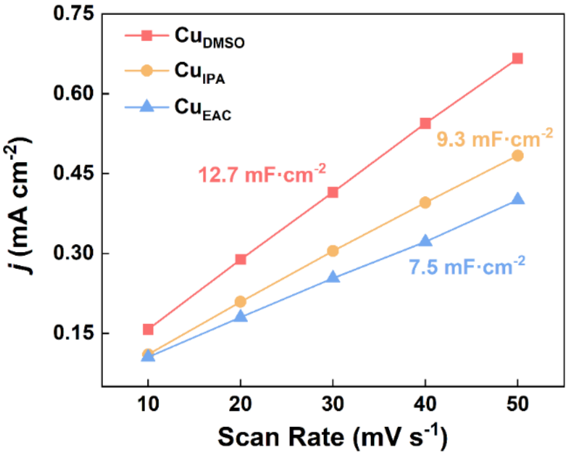

Figure S18. Plot of difference in current density vs. scan rate.

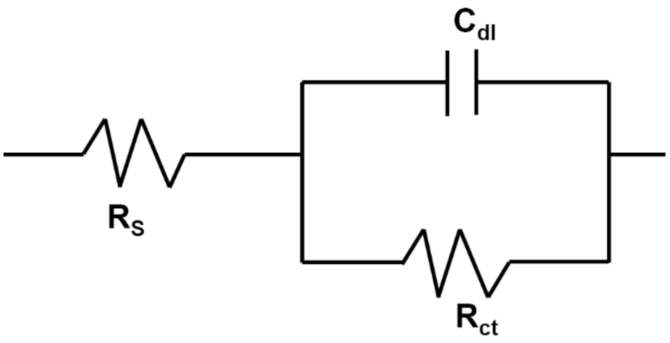

Figure S19. Electrical equivalent circuit used for simulating the EIS data.

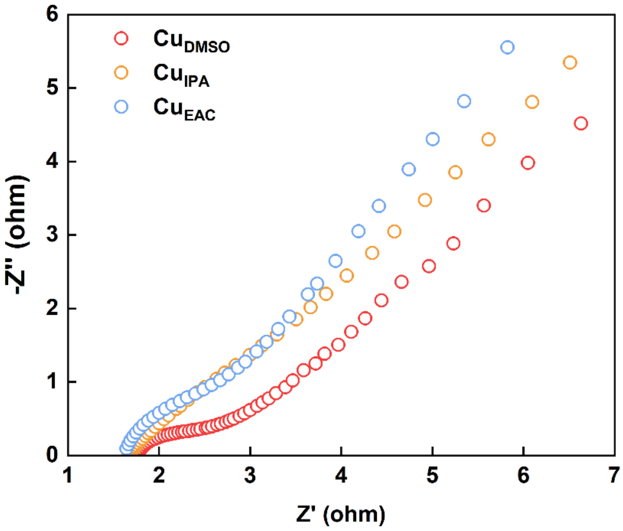

Figure S20. Nyquist plots using different electrodes.

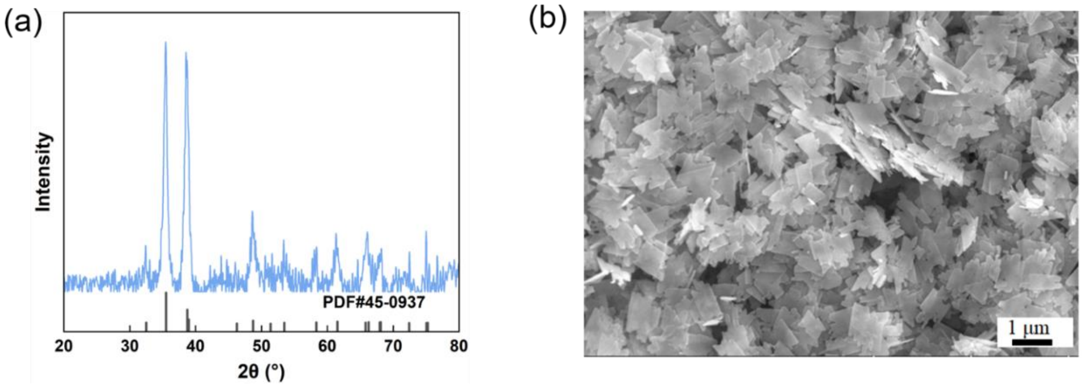

Figure S21. XRD pattern (a) and SEM image (b) of CuO-NS.

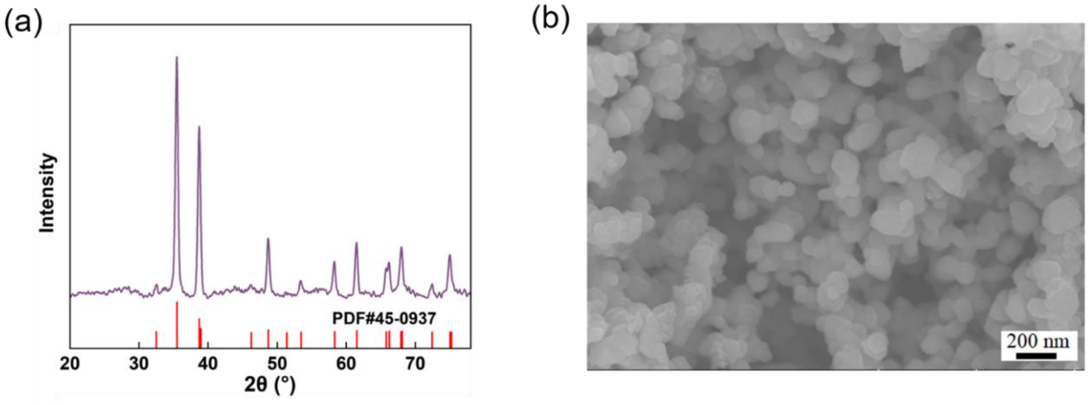

Figure S22. XRD pattern (a) and SEM image (b) of CuO-NP.

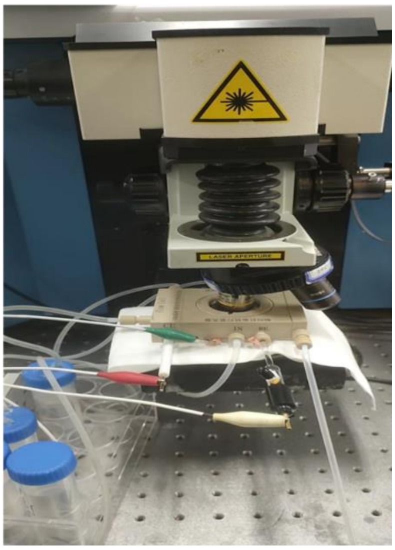

Figure S23. The in situ electrochemical spectral cell for Raman test.

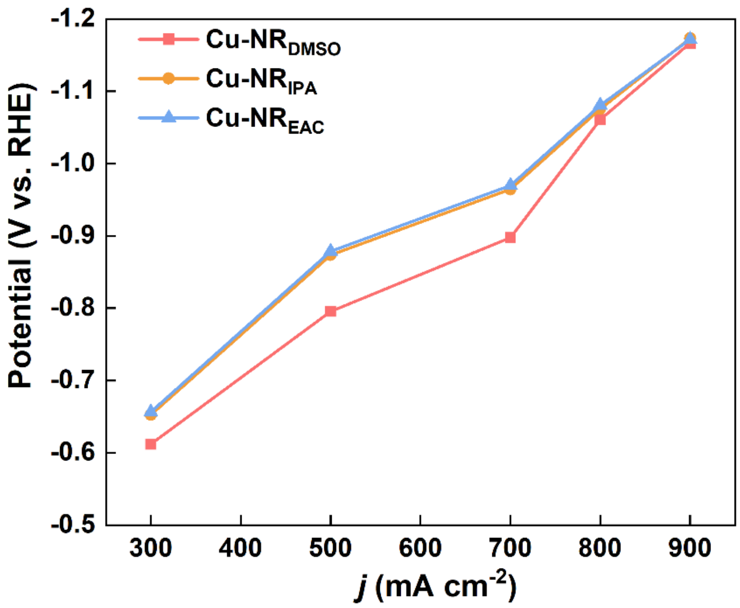

Figure S24. Plot of potential vs. current density over different electrodes.

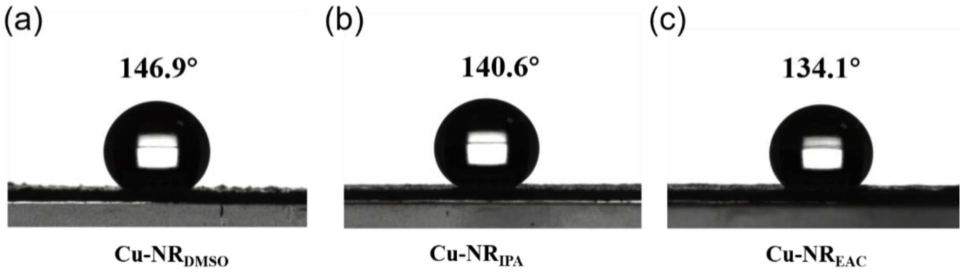

Figure S25. Contact angles between surfaces of CuO-NR electrodes and water droplet.

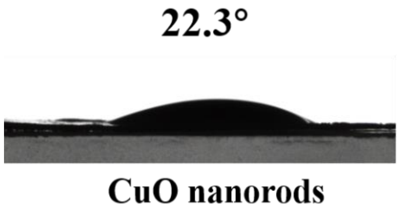

Figure S26. Contact angles between surfaces of CuO-NR and water droplet.

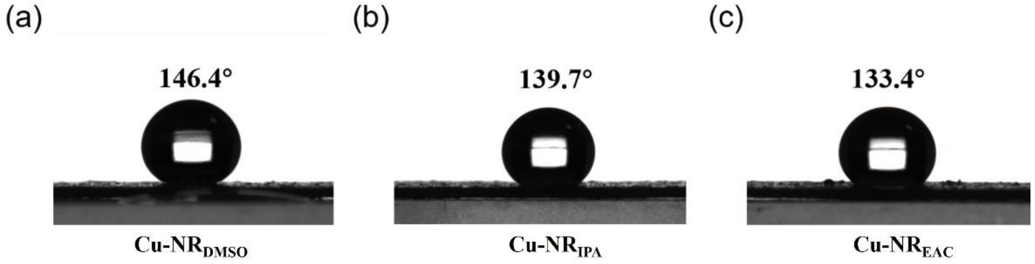

Figure S27. Contact angles between surfaces of Cu-NR electrodes and water droplet after 24 hours of electrolysis.

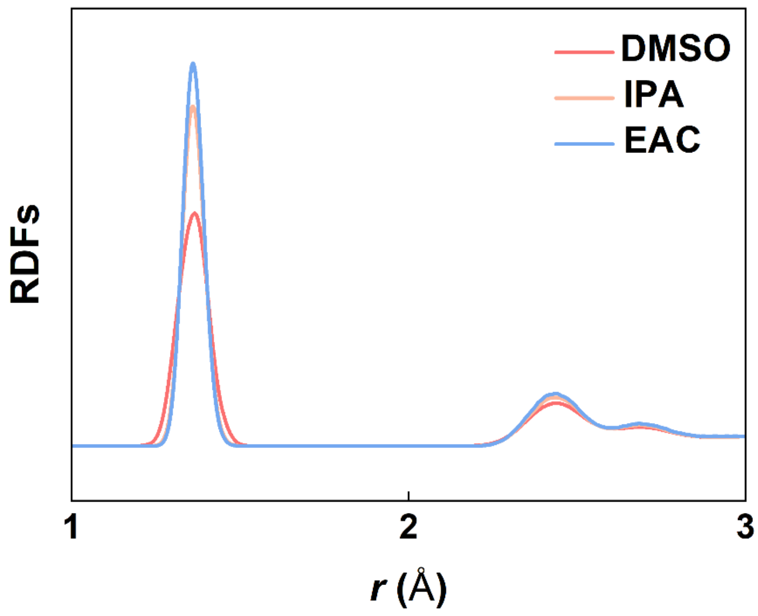

Figure S28. RDFs of B–S in different solvents.

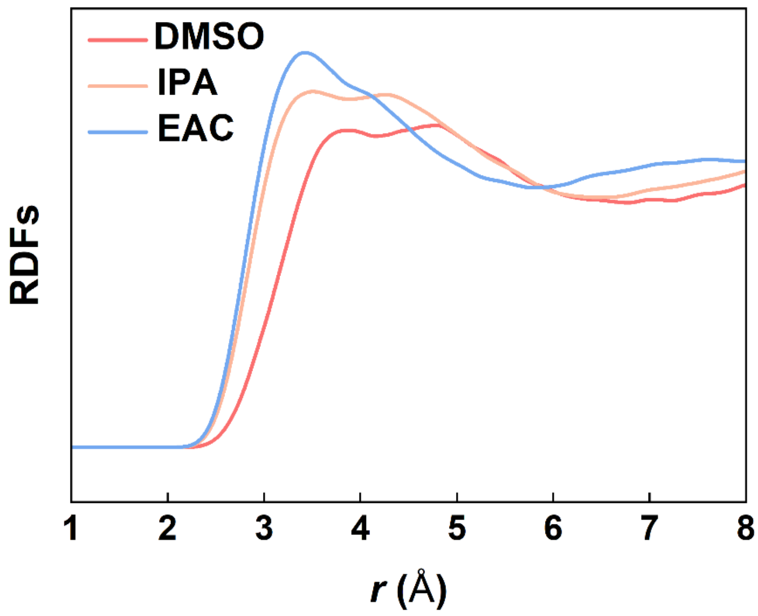

Figure S29. RDFs of Cu–B in different solvents.

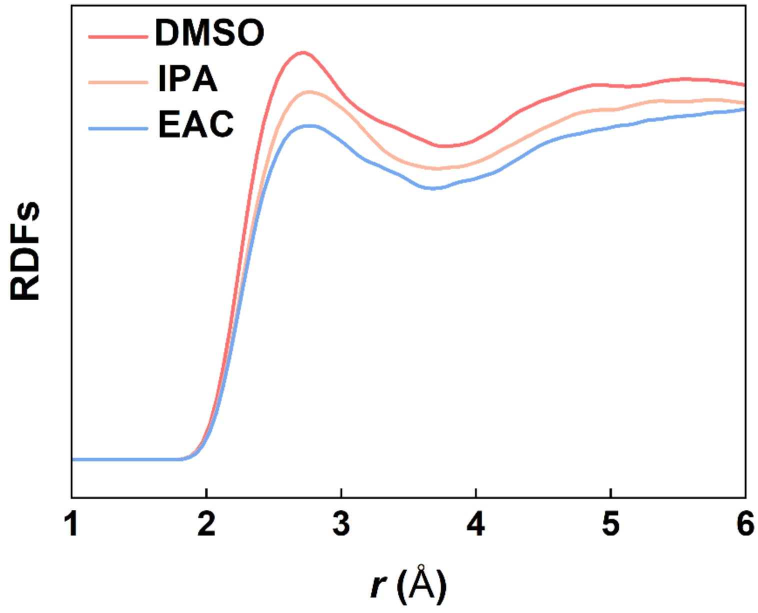

Figure S30. RDFs of Cu–S in different solvents.

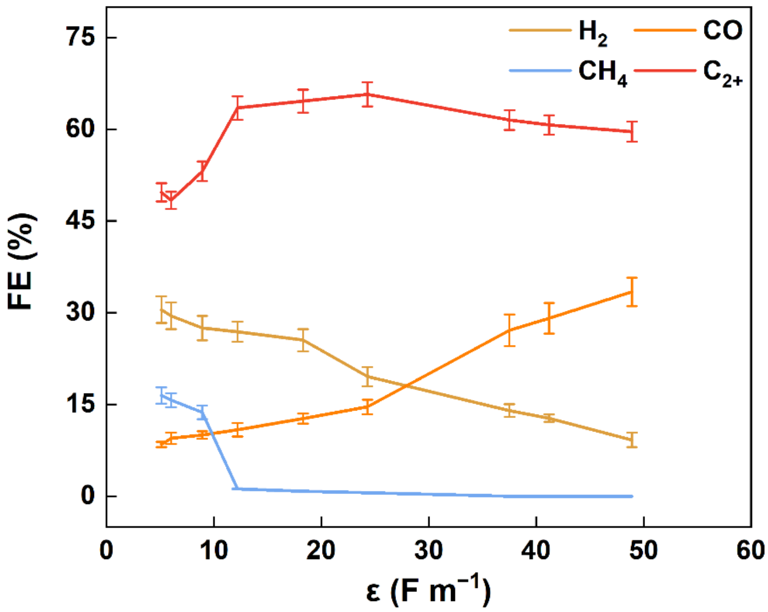

Figure S31. FEs of different products over various Cu-NR electrodes prepared in solvents with varying ε values at 800 mA cm^{-2}.

Supplementary Tables

Table S1. The abbreviation and ε values of different solutions.

Solutions	Abbreviation	ε (F m^{-1})
Chloroform	TCM	5.1
Ethyl acetate	EAC	6.02
Dichloromethane	MC	8.9
Butanol	TBA	12.5
2-propanol	IPA	18.3
Ethanol	EA	24.3
Ethylene glycol	EG	37.7
2-furaldehyde	FD	41.9
Dimethyl sulfoxide	DMSO	48.9
