# Supplementary material for: Modulating CO2 electroreduction pathways through controlled ionomer arrangement on catalyst surfaces via solvent dispersion
Source: Innovation (Camb). 2025 Mar 18;6(6):100882. doi: 10.1016/j.xinn.2025.100882 (PMC12169282; doi:10.1016/j.xinn.2025.100882)
Supplement: Document S2. Article plus supplemental information [file mmc2.pdf]

OPEN ACCESS

Modulating CO_2 electroreduction pathways through controlled ionomer arrangement on catalyst surfaces via solvent dispersion

Yaoyu Yin,[1,2] Zhongnan Ling,[3,4] Shiqiang Liu,[1] Jiapeng Jiao,[5] Meng Zhou,[1] Pei Zhang,[1] Xing Tong,[1,2] Yueqian Fan,[3] Jiahao Yang,[1,2] Huanyan Liu,[3] Xueqing Xing,[3] Jianling Zhang,[1,2] Yi Xu,[1,2] Hongyan Liang,[6] Xinchen Kang,[1,2,*] and Buxing Han[1,2,5,*]
*Correspondence: kangxinchen@iccas.ac.cn (X.K.); hanbx@iccas.ac.cn (B.H.)

Received: September 11, 2024; Accepted: March 13, 2025; Published Online: March 18, 2025; https://doi.org/10.1016/j.xinn.2025.100882

© 2025 The Author(s). Published by Elsevier Inc. on behalf of Youth Innovation Co., Ltd. This is an open access article under the CC BY license (http://creativecommons.org/licenses/by/4.0/).

GRAPHICAL ABSTRACT

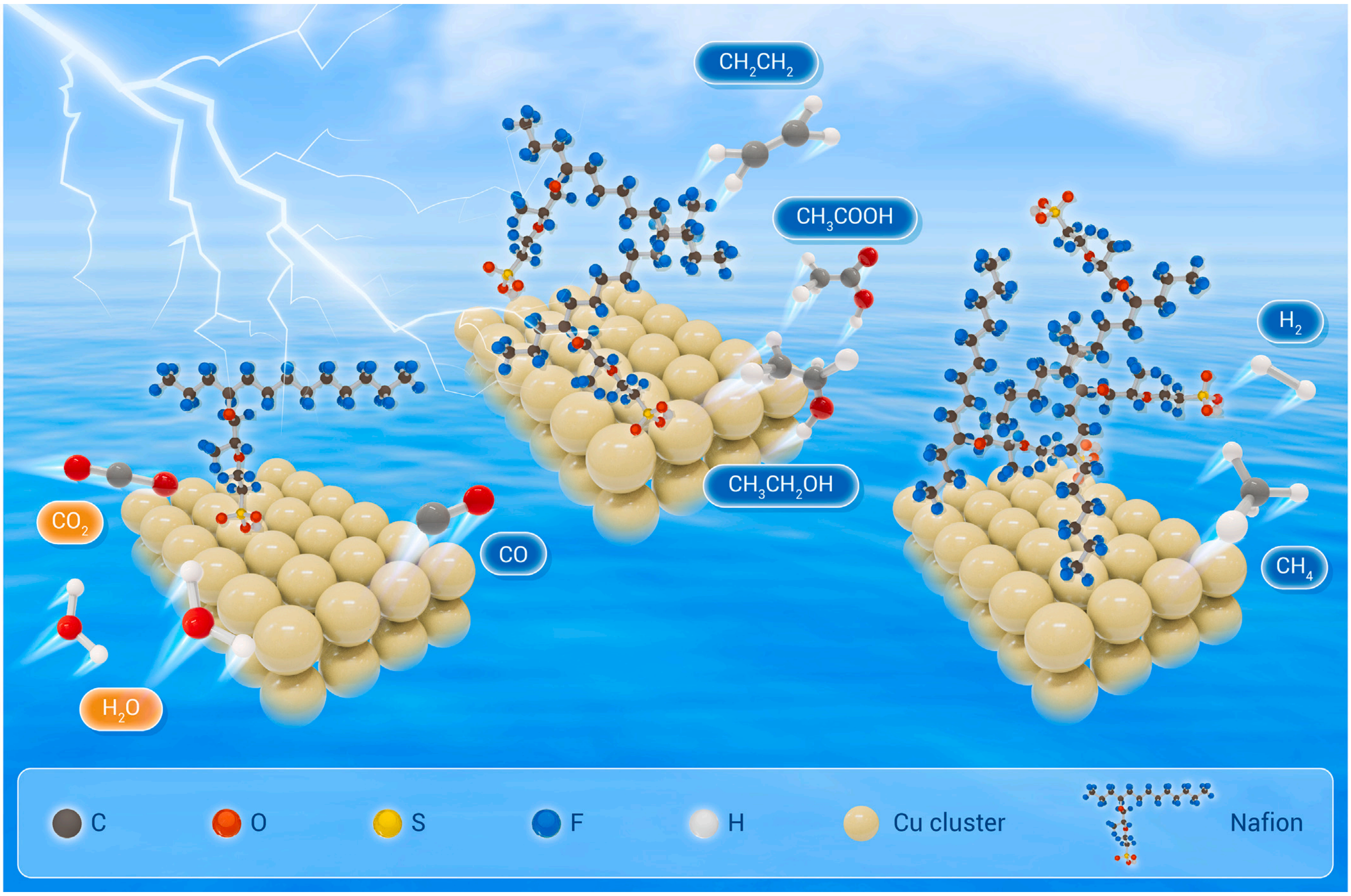

PUBLIC SUMMARY

- A universal and novel approach for fine-tuning the product distribution of CO_2 electroreduction is presented.
- The dispersion solvent of the catalyst ink influences ionomer configuration.
- Ionomer configuration within the catalyst layer affects the binding of intermediates in CO_2 electroreduction.
- Faradaic efficiency for multicarbon products can reach 90.5% at a current density of 800 mA cm^{-2}.

Check for updates

OPEN ACCESS

REPORT

Modulating CO_2 electroreduction pathways through controlled ionomer arrangement on catalyst surfaces via solvent dispersion

Yaoyu Yin,[1,2] Zhongnan Ling,[3,4] Shiqiang Liu,[1] Jiapeng Jiao,[5] Meng Zhou,[1] Pei Zhang,[1] Xing Tong,[1,2] Yueqian Fan,[3] Jiahao Yang,[1,2] Huanyan Liu,[3] Xueqing Xing,[3] Jianling Zhang,[1,2] Yi Xu,[1,2] Hongyan Liang,[6] Xinchen Kang,[1,2,*] and Buxing Han[1,2,5,*]

[1]CAS Laboratory of Colloid and Interface and Thermodynamics, Institute of Chemistry, Chinese Academy of Sciences, Beijing 100190, China
[2]School of Chemistry and Chemical Engineering, University of Chinese Academy of Sciences, Beijing 100049, China
[3]Beijing Synchrotron Radiation Facility, Institute of High Energy Physics, Chinese Academy of Sciences, Beijing 100049, China
[4]College of Materials Science and Engineering, Shenyang University of Chemical Technology, Shenyang 110142, China
[5]School of Chemistry and Molecular Engineering, East China Normal University, Shanghai 200062, China
[6]School of Materials Science and Engineering and Key Laboratory of Efficient Utilization of Low and Medium Grade Energy, Tianjin University, Tianjin 300350, China
*Correspondence: kangxinchen@iccas.ac.cn (X.K.); hanbx@iccas.ac.cn (B.H.)

Received: September 11, 2024; Accepted: March 13, 2025; Published Online: March 18, 2025; https://doi.org/10.1016/j.xinn.2025.100882

© 2025 The Author(s). Published by Elsevier Inc. on behalf of Youth Innovation Co., Ltd. This is an open access article under the CC BY license (http://creativecommons.org/licenses/by/4.0/).

Citation: Yin Y., Ling Z., Liu S., et al., (2025). Modulating CO_2 electroreduction pathways through controlled ionomer arrangement on catalyst surfaces via solvent dispersion. The Innovation **6(6)**, 100882.

Ionomers play a vital role in the preparation of electrodes for CO_2 electroreduction, and controlling the ionomer configuration on the catalyst surface offers an effective strategy for adjusting the surface microenvironment of the electrode, thereby influencing the distribution of CO_2 electroreduction products. In this study, we demonstrate that Nafion, a commonly used ionomer, exhibits distinct aggregation behaviors in solvents with different dielectric constant (ε) values. These differences in aggregation result in varied Nafion arrangements on the catalyst surface, which in turn affect the binding of *CO and *H intermediates, enabling control over product distribution. For example, over a Cu nanosheet catalyst at 800 mA cm^{-2}, the Faradaic efficiency for multicarbon products increases from 67.5% to 90.5% simply by changing the dispersion solvent from low-ε dimethyl sulfoxide to moderate-ε isopropanol. This work introduces a novel approach for fine-tuning CO_2 electroreduction product distribution through manipulation of the dispersion solvent without requiring modifications to the catalyst or ionomer.

INTRODUCTION

The electrochemical CO_2 reduction reaction (CO_2RR) presents a promising route for synthesizing high-value chemicals.[1–3] Cu-based materials have emerged as efficient catalysts for converting CO_2 into versatile products.[4–6] Fine-tuning the microenvironment of the Cu surface to control the adsorption of *CO and *H intermediates is crucial for determining CO_2RR selectivity, as these intermediates play pivotal roles in C–C coupling and the hydrogen evolution reaction (HER), respectively.[7–9] Modulating the electrode surface microenvironment to control *CO adsorption is particularly effective in steering the CO_2RR pathway. Various strategies, including local pH regulation, surface field tuning, and selective intermediate stabilization, have been explored to tailor the reactivity and product distribution of Cu electrodes.[10–15] Ionomers are commonly incorporated into electrodes to enhance catalyst dispersion and facilitate proton and electron transfer during the CO_2RR. Moreover, ionomers influence the microenvironment at the electrode surface, thereby affecting the intermediate adsorption behavior and ultimately altering CO_2RR product distribution.[16–18]

Nafion, the primary ionomer used in CO_2RR electrode fabrication, comprises a polytetrafluoroethylene $-(CF_2)_n-$ backbone with a perfluorinated vinylpolyether side chain containing sulfonic acid terminal groups ($-SO_3H$).[19,20] The monomer structure of Nafion is shown in Figure S1. The sulfonic acid groups exhibit a strong affinity for material surfaces, while the $-(CF_2)_n-$ backbone exhibits notable hydrophobicity.[21–23] These characteristics influence the configuration of Nafion molecules on the catalyst surface, thereby modulating the electrode surface microenvironment and enabling tunable *CO and *H adsorption during the CO_2RR.[24] Although previous research has investigated how dispersion solvents influence ionomer-catalyst interactions and, consequently, the morphology and structure of electrodes,[25] no systematic study has been conducted on tailoring the Nafion configuration on the electrode surface through solvent selection. The dielectric constant (ε) of a solvent, which reflects its polarity, strongly influences the aggregation behavior of Nafion.[21] This aggregation behavior, in turn, affects the arrangement of Nafion molecules on the catalyst surface, ultimately impacting CO_2RR performance.

Herein, we used various solvents to disperse the catalyst and Nafion, resulting in electrodes with differing Nafion arrangements on the catalyst surface. This variation influences the adsorption of key CO_2RR intermediates, leading to different product distributions. For example, solely changing the dispersion solvent alters the Faradaic efficiency (FE) ratio of CO to multicarbon (C_{2+}) products, ranging from 0.5 to 2.5 over a Cu nanorod (Cu-NR) electrode. Over a Cu nanosheet (Cu-NS) electrode, FE_{C2+} increases from 67.5% to 90.5% at 800 mA cm^{-2} by changing the dispersion solvent from dimethyl sulfoxide (DMSO) to isopropanol (IPA). A comprehensive investigation involving *in situ* surface-enhanced Raman spectroscopy (SERS), *in situ* attenuated total reflection surface-enhanced infrared absorption spectroscopy (ATR-SEIRAS), molecular dynamics (MD) simulations, dynamic light scattering (DLS), small-angle X-ray scattering (SAXS), and cryoelectron transmission electron microscopy (cryo-TEM) was conducted to elucidate the underlying mechanism.

MATERIALS AND METHODS

Materials

$CuSO_4\cdot 5H_2O$, $C_6H_5O_7Na_3\cdot 2H_2O$, NaOH, Nafion D-521 dispersion (5 wt %) and anion exchange membrane (FumasepFAA-3-PK-130), and hydrophobic carbon paper (CP) were purchased from Alfa Aesar China. KOH, DMSO, IPA, and ethyl acetate (EAC) were obtained from Aladdin Reagents (Shanghai). CO_2 (99.999%) and CO (99.999%) were provided by Beijing Analytical Instrument Company. Commercial Cu nanoparticles purchased from Sigma-Aldrich were used.

Synthesis of Cu-NR electrodes

1.3 mmol $CuSO_4\cdot 5H_2O$ and 0.91 mmol $C_6H_5O_7Na_3\cdot 2H_2O$ were dissolved in 40 mL of deionized water. After stirring for 15 min at room temperature, 5.3 mmol of NaOH was added into the solution and stirred for another 2.5 h. The resultant mixture was then transferred into an autoclave and heated at 160°C for 12 h. Upon completion of the reaction, the mixture was separated, washed by deionized water and ethanol 5 times, and dried *in vacuo* at 60°C for 8 h. The solid was annealed at 400°C for 4 h with a heating rate of 10°C/min, and CuO-NR was obtained. 10 mg of the as-prepared CuO-NR and 30 μL of Nafion solution were dispersed into 1 mL of solvent and sonicated for 30 min to obtain a homogeneous catalyst ink. The catalyst ink was loaded onto a hydrophobic CP (2 × 1.5 cm) and dried under an infrared lamp for 5 min to obtain the gas diffusion electrode. The amount of catalyst on the electrode surface was ~1.0 mg cm^{-2}. Finally, the as-prepared CuO-NR electrodes were electroreduced in 1 M KOH at −0.5 V vs. RHE for 10 min, resulting in Cu-NR electrodes denoted as $Cu\text{-}NR_{DMSO}$, $Cu\text{-}NR_{IPA}$, and $Cu\text{-}NR_{EAC}$, respectively.

Synthesis of Cu-NS electrodes

$CuCl_2$ aqueous solution (0.12 M, 30 mL) was added into a 3 M NaOH solution of 30 mL, and the solution was vigorously stirred for 30 min. Then, the solution was transferred into an autoclave and heated at 100°C for 12 h. Upon completion of the reaction, the mixture was separated, washed by deionized water and ethanol 5 times, and dried *in vacuo* at 60°C for 8 h, and CuO-NS was obtained. 10 mg of the as-prepared CuO-NS and 30 μL of Nafion solution were dispersed in 1 mL of solvent and sonicated for 30 min to obtain a homogeneous catalyst ink. The catalyst ink was loaded onto a hydrophobic CP (2 × 1.5 cm) and dried under an infrared lamp for 5 min to obtain the gas diffusion electrode. The amount of catalyst on the electrode surface was ~1.0 mg cm^{-2}. Finally, the as-prepared CuO-NS electrodes were electroreduced in 1 M KOH at −0.5 V vs. RHE for 10 min, resulting in Cu-NS electrodes denoted as $Cu\text{-}NS_{DMSO}$, $Cu\text{-}NS_{IPA}$, and $Cu\text{-}NS_{EAC}$, respectively.

Synthesis of Cu-NP electrodes

10 mg of the commercial CuO and 30 μL of Nafion solution were dispersed in 1 mL of solvent and sonicated for 30 min to obtain a homogeneous catalyst ink. The catalyst ink was loaded onto CP (2 × 1.5 cm) and dried under an infrared lamp for 5 min to obtain the gas diffusion electrode. The amount of catalyst on the electrode surface was ~1.0 mg cm^{-2}. Finally, the as-prepared CuO-NP electrodes were electroreduced in 1 M KOH at −0.5 V vs. RHE for 10 min, resulting in Cu-NP electrodes denoted as Cu-NP_{DMSO}, Cu-NP_{IPA}, and Cu-NP_{EAC}, respectively.

Characterizations

DLS was performed on ALV CGS-3 at room temperature. SAXS experiments were performed at Beamline 1W2A of the Beijing Synchrotron Radiation Facility (BSRF). Cryo-TEM (Thermo Fisher Scientific Themis 300) was used to characterize the specific morphology of Nafion in different dispersions. To ensure that the ionomer structures remained undamaged under electron exposure, we used electron doses below these limits for imaging: 4–5 $e^-/Å^2$ for EAC, 7–8 $e^-/Å^2$ for IPA, and 20 $e^-/Å^2$ for DMSO. ^{19}F-nuclear magnetic resonance (NMR) study was performed using a Bruker Avance III 400 HD spectrometer. X-ray diffraction (XRD) was conducted on an X-ray diffractometer (Model D/MAX2500, Rigaka) using a Cu-Kα source. Scanning electron microscopy (SEM) images were captured on SEM JEOL SU8020. The surface altitude and roughness were obtained on an AFM MultiMode8 and Optical profilometer ContourGT-K1. The conductivities were determined by a CGS-MT mini multi-functional probe station (Sino Aggtech, Beijing). X-ray absorption spectroscopy measurements were performed at Beamline 1W2B of BSRF. The energy was tuned by an Si (111) monochromator. The data were collected in fluorescence excitation mode using a Lytle detector. *In situ* Raman spectra were conducted on LabRAM HR Evolution using a 785 nm solid laser as an excitation source in 1 M KOH. *In situ* ATR-SEIRAS was recorded on VERTEX 70v in 0.5 M $KHCO_3$. The static contact angles were measured using an OCA20 apparatus (Data-Physics, Germany). All measurements were repeated five times, and the error range for all samples is consistently within 1°–2°. The Arrhenius equation was used to calculate the activation energy of the proton migration: $\sigma = \sigma_o \exp(Ea/kT)$, where σ_o is the pre-exponential factor, Ea is the activation energy, k is the Boltzmann constant, and T is the absolute temperature (in K).

Electrochemical experiments

All the electrochemical experiments were conducted on the electrochemical workstation (CHI 660E). 10 mg of catalyst was dispersed into 1 mL of solvent with 30 μL of Nafion solution and sonicated for 30 min to obtain a homogeneous catalyst ink. Subsequently, the catalyst ink was loaded onto CP (2 × 1.5 cm) and dried under an infrared lamp for 5 min to obtain the gas diffusion electrode. The amount of catalyst on the electrode surface was ~1.0 mg cm^{-2}. An anion exchange membrane (FumasepFAA-3-PK-130) was used to separate the anodic and cathodic chambers. A nickel plate coated with iridium oxide was used as the anode. An Hg/HgO electrode was used as the reference electrode, and it was calibrated with respect to RHE: E (vs. RHE) = E (vs. Hg/HgO) + 0.098 V + 0.0591 V × pH and compensated with the solution resistance. Prior to the reaction, all electrodes were electroreduced in 1 M KOH at −0.5 V vs. RHE for 10 min. The CO_2RR was performed by constant current electrolysis in 1 M KOH in a flow cell. After the reaction, gaseous products were collected using a gas bag and analyzed by gas chromatography, and liquid products were measured by ^{1}H-NMR spectroscopy. Electrochemical impedance spectroscopy (EIS) measurement was carried out in 1 M KOH solution with an amplitude of 5 mV and a frequency from 10^{-1} to 10^5 Hz. Double-layer capacitance (C_{dl}) values were obtained by cyclic voltammogram (CV) scanning performed in the non-Faraday zone with different scan rates in 1 M Ar-saturated KOH solution.

CO stripping experiments were carried out using 0.1 M $KHCO_3$ as the electrolyte in an H-type cell. Prior to the experiment, all catalysts were electrolyzed at −0.6 V vs. RHE for 5 min to fully remove the oxidation species in 0.1 M Ar-saturated $KHCO_3$. CO was then introduced into the cell and electrolyzed at −0.8 V vs. RHE for 10 min to obtain CO adsorption at the cathode. Ar was then flowed into the electrolyte to remove residual CO. CV curves were then conducted at a scan rate of 50 mV s^{-1}.

Electrochemical OH^- adsorption over the CuO electrode was performed in a 1 M Ar-saturated KOH electrolyte using linear sweep voltammetry at a sweep rate of 100 mV s^{-1}. Prior to the experiment, all catalysts were electrolyzed at −0.6 V vs. RHE for 5 min to fully remove the oxidation species.

DFT simulations

Spin-polarized density functional theory (DFT)[26] is conducted in the Vienna Ab initio Simulation Package (VASP).[27] We adopt projected augmented-wave (PAW) method[28] potentials and the exchange-correlation energy of the general gradient approximation (GGA) in the scheme proposed by Perdew-Burke-Ernzerh (PBE).[29] The cutoff kinetic energies for the plane waves are set to 450 eV for all the calculations. The convergence tolerance values of the energy and force on each atom during structure relaxation are less than 10^{-5} eV and 0.05 eV $Å^{-1}$, respectively. The system is too large and includes more than 100 atoms; thus, a set of Monkhorst-Pack mesh K points of gamma point is used to sample the Brillouin zone for geometry optimization.[30] The c axis is set to 15 Å to ensure sufficient vacuum to avoid interactions between the two periods.[31] Grimme's DFT-D3 scheme was used to describe the van der Waals (vdW) interactions in the systems.[32] The catalyst Cu is modeled by a three-atomic-layer slab with a 6 × 6 in-plane supercell (108 atoms) and exposed (111) facets, and the lattice parameters for the structure studied in this work are a = 15.36 Å, b = 13.28 Å, c = 30.00 Å, and $\alpha = \beta = \gamma = 90°$.

The adsorption energy (E_{ads}) of *CO is determined using the following equation:

$$E_{ads} = E_{(total)} - E_{(surface)} - E_{(CO)}$$

MD simulations

MD simulations were performed using GROMACS software package.[33] The initial analog box size was 60 × 60 × 60 nm^3. It included water (400), Nafion (70), solvent (800), and CuO (300). It should be noted that in our MD simulations, we employed Nafion monomers rather than Nafion itself. This is a commonly used strategy to study ionomers.[34] The particle mesh Ewald method[35] with a precision of 1 × 10^{-6} was used to calculate the long-range electrostatic interactions.[36] The systems were firstly heated from 298 to 500 K for 1 ns and maintained at 500 K for 2 ns and then subsequently annealed from 500 to 298 K for 1 ns and then 298 K for 2 ns under the isothermal-isobaric NPT (constant-pressure, constant-temperature) ensemble using a velocity-rescale thermostat and Berendsen barostat with a relaxation constant of 1.0 ps each. Dynamics trajectories were collected for subsequent data analysis, which was performed at 298 K and 1 atm for 500 ps under the isothermal-isometric NVT ensemble using a velocity-rescale thermostat with a time step of 1.0 fs.[37]

RESULTS

CO_2RR over different electrodes

CuO-NR catalysts were first prepared; the corresponding characterization results, including XRD patterns and SEM images, are shown in Figures S2 and S3. DMSO, IPA, and EAC, with high-, moderate-, and low-ε values of 48.9, 18.3, and 6.02, respectively, were selected as representative solvents for dispersing CuO-NR catalysts and Nafion to prepare CuO-NR electrodes. The CuO-NR electrodes were subsequently electroreduced to form Cu-NR electrodes (Figure S4). Cu-NR electrodes prepared in DMSO, IPA, and EAC are denoted Cu-NR_{DMSO}, Cu-NR_{IPA}, and Cu-NR_{EAC}, respectively. All electrodes exhibit identical polycrystalline copper structures (Figure S5), rod-like morphologies (Figure S6), and Cu(0) states (Figure S7), as confirmed by XRD patterns, SEM images, and Cu K-edge X-ray absorption near-edge structure (XANES) spectra. While Cu-NR_{DMSO} and Cu-NR_{IPA} exhibit uniform morphologies (Figure S8), Cu-NR_{EAC} displays pronounced particle agglomeration. SEM and atomic force microscopy (AFM) images reveal nonuniform thickness across all electrodes, with Cu-NR_{EAC} exhibiting the highest roughness (Figures S9 and S10). The order of surface roughness is Cu-NR_{DMSO} < Cu-NR_{IPA} < Cu-NR_{EAC}, as demonstrated by three-dimensional (3D) profiling images (Figure S11), consistent with the AFM results. The surface roughness of Cu-NR electrodes is similar to that of CuO-NR electrodes (Figures S12 and S13), indicating that the electroreduction process does not alter the surface morphology. Conductivity-temperature relationship analysis, following the Arrhenius equation,[38] reveals proton migration activation energies of 4.76 (Cu-NR_{DMSO}), 6.23 (Cu-NR_{IPA}), and 9.02 (Cu-NR_{EAC}) kJ mol^{-1}, indicating that electrodes prepared in high-ε solvents favor proton migration (Figure S14).

The catalytic performance of the aforementioned electrodes for the CO_2RR was evaluated through constant current density electrolysis in 1 M KOH in a flow cell (Figure S15). Across all electrodes, FE_{H2} increases, FE_{CO} decreases, and FE_{C2+} initially increases and subsequently decreases with increasing current density (Figures 1A–1C). However, electrodes obtained from different inks display distinct product distributions, particularly in C_{2+} vs. CO. For instance, at j = 800 mA cm^{-2}, Cu-NR_{DMSO}, Cu-NR_{IPA}, and Cu-NR_{EAC} exhibit FE_{C2+} values of 55.6%, 64.6%, and 44.2%, respectively, and FE_{CO} values of 29.4%, 8.6%, and 9.4%, respectively. Furthermore, FE_{CH4} reaches 15.7% over Cu-NR_{EAC} but remains negligible over Cu-NR_{DMSO} and Cu-NR_{IPA} (Figure 1D). The FE_{CO}/FE_{C2+} ratio exceeds 1 over Cu-NR_{DMSO} but falls below 1 over Cu-NR_{IPA} and Cu-NR_{EAC} at j < 700 mA cm^{-2}. Notably, at j = 300 mA cm^{-2}, the FE_{CO}/FE_{C2+} ratio is ~2.5 over Cu-NR_{DMSO} but <0.5 over Cu-NR_{EAC} (Figure 1E). The order of

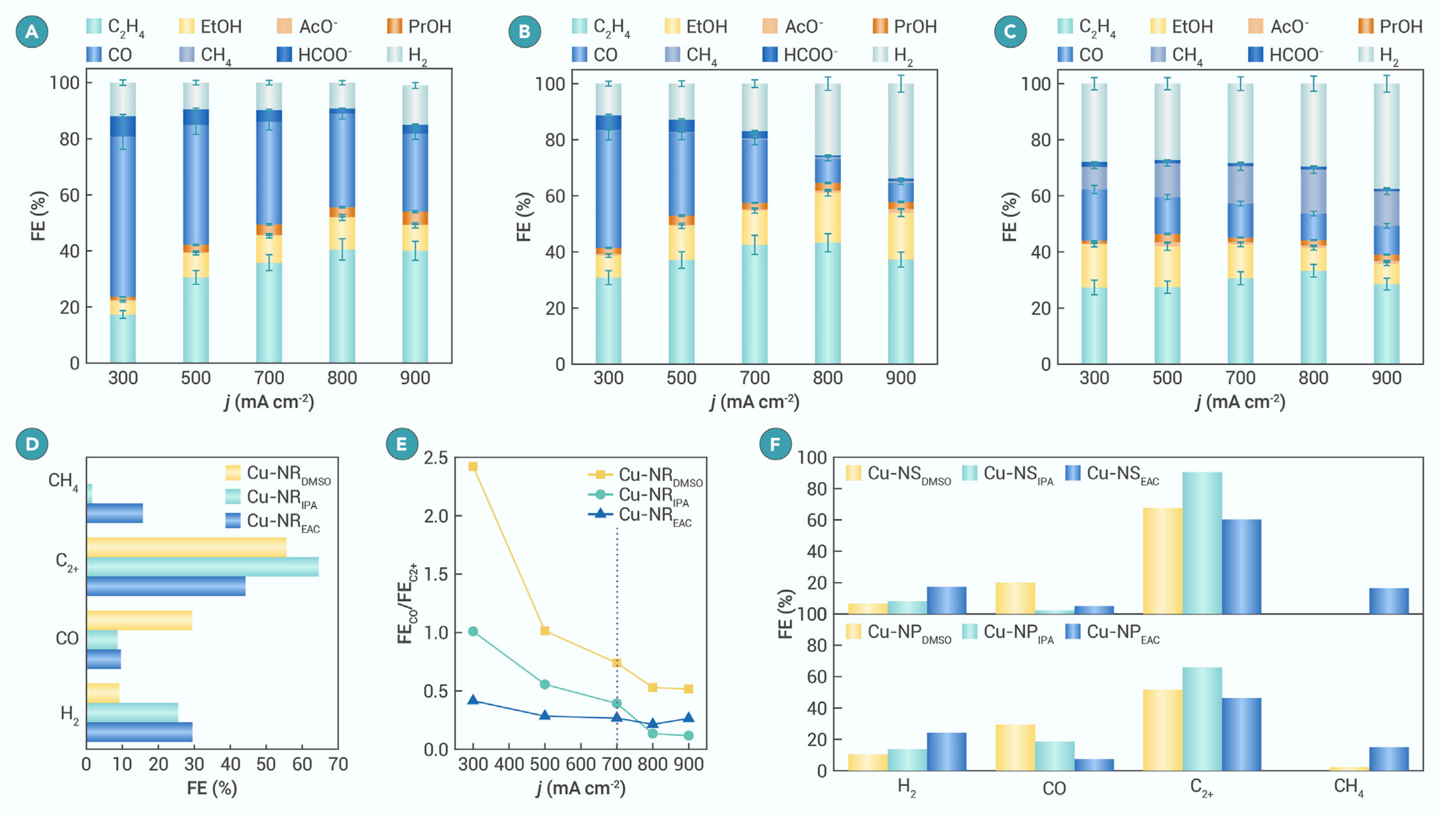

Figure 1. CO_2RR in 1 M KOH (A–C) Plot of FEs of different products vs. current density over Cu-NR$_{DMSO}$ (A), Cu-NR$_{IPA}$ (B), and Cu-NR$_{EAC}$ (C). (D) FEs of different products over various Cu electrodes at j = 800 mA cm^{-2}. (E) Plot of FE$_{CO}$/FE$_{C2+}$ vs. current density over various Cu electrodes. (F) FEs of different products over Cu-NS and Cu-NP catalysts at j = 800 mA cm^{-2}.

FE_{H_2} is Cu-NR$_{EAC}$ > Cu-NR$_{IPA}$ > Cu-NR$_{DMSO}$, indicating simultaneous promotion of C–C coupling and the HER over electrodes prepared in low-ε solvents. Cu-NR$_{IPA}$ exhibits the highest FE$_{C2+}$, attributable to moderate CO and H_2 production. After 24 h of continuous electrolysis at 800 mA cm^{-2}, FE$_{C2+}$ remains nearly constant across all electrodes, demonstrating their stability (Figure S16). Cu-NR$_{EAC}$ exhibits the lowest C_{dl} and the highest charge transfer resistance (R_{ct}), as deduced from the fitting of CV curves and Nyquist plots (Figures S17–S20),[39] indicating a reduced active surface area and some hindered charge transfer due to catalyst agglomeration. Cu-NS and Cu-NP electrodes prepared using various solvents were also applied for the CO_2RR; the corresponding characterization results, including XRD patterns and SEM images, are shown in Figures S21 and S22. Similar trends were observed: electrodes prepared in high-ε solvents favor CO production, while those prepared in low-ε solvents promote C–C coupling and the HER (Figure 1F). For instance, FE$_{C2+}$ increases from 67.5% to 90.5% over the Cu-NS catalyst at 800 mA cm^{-2} solely by changing the ink solvent from DMSO to IPA. These results indicate that changing the solvent used for dispersing the catalyst to prepare electrodes is a universal and effective strategy for directing the selectivity of the CO_2RR.

In situ characterization of CO_2RR intermediates

In situ SERS was conducted to characterize CO_2RR intermediates (Figures 2A–2C and S23).[40–42] A peak at 510 cm^{-1}, attributed to adsorbed CO_2, is observed over Cu-NR$_{DMSO}$ but is absent over Cu-NR$_{IPA}$ and Cu-NR$_{EAC}$. This suggests that Cu-NR$_{DMSO}$ creates a CO_2-rich environment, consequently requiring less negative potential to drive the CO_2RR (Figure S24). The Raman peak at 370 cm^{-1}, assigned to Cu–CO stretching, reflects the adsorption of *CO intermediates on the catalyst surface.[43–45] The strength of *CO adsorption correlates with the formation of CH_4, C_{2+}, and CO, representing strong, moderate, and weak adsorption, respectively.[46,47] Integration of the Cu–CO Raman peak area across different electrodes indicates that the *CO coverage on Cu-NR$_{IPA}$ is considerably higher than that on Cu-NR$_{DMSO}$ (Figure 2D). CO stripping test results reveal that the order of CO adsorption strength is Cu-NR$_{EAC}$ > Cu-NR$_{IPA}$ > Cu-NR$_{DMSO}$ (Figure 2E), aligning with the observed carbonaceous product distributions (Figures 1D–1F). In addition, a Cu–OH Raman peak at 700 cm^{-1}, with the highest intensity observed for Cu-NR$_{IPA}$, indicates the highest local OH$^-$ concentration on the Cu-NR$_{IPA}$ surface (Figure 2F),[48,49] consistent with the Raman analysis results. Adsorbed OH$^-$ contributes to the formation of a locally alkaline microenvironment, which facilitates C–C coupling and thus enhances C_{2+} product formation,[50] ultimately leading to the highest FE$_{C2+}$ over Cu-NR$_{IPA}$. Further confirmation of the enhanced C–C coupling capability of Cu-NR$_{IPA}$ is provided by the *in situ* ATR-SEIRAS results, where more intense peaks at 1,390 and 1,200 cm^{-1}, corresponding to *COOH and *OCCOH intermediates, respectively, are observed (Figures 2G–2I).

Plausible mechanism

Cu electrodes prepared using different solvents exhibit diverse product distributions during the CO_2RR. Because all Cu electrodes share identical composition and structure, the variation in product distribution can be attributed solely to the arrangement of Nafion on the Cu surface. The Raman peak at 1,128 cm^{-1}, corresponding to sulfonic acid groups (δ(Cu–SO_3H)) adsorbed on Cu surfaces,[34,51] is the most intense for Cu-NR$_{DMSO}$ but is notably attenuated for Cu-NR$_{EAC}$ (Figure 3A). This indicates that –SO_3H groups in Nafion preferentially adsorb onto Cu-NR$_{DMSO}$, while the hydrophobic –$(CF_2)_n$– backbone extends outward from the electrode surface. DFT calculations were performed to elucidate the adsorption mechanism of Nafion on Cu surfaces during the CO_2RR. The results indicate that strong adsorption of –SO_3H groups hinders *CO intermediate adsorption (Figures 3B–3E), thereby suppressing C–C coupling over Cu-NR$_{DMSO}$. Contact angle measurements show that Cu-NR electrodes exhibit contact angles that are comparable with those of CuO-NR electrodes (Figures 3F–3H and S25); however, these contact angles are significantly higher than those observed for the bare CuO-NR sample (Figure S26). This confirms that Nafion plays a crucial role in regulating the surface hydrophobicity of the electrodes. The contact angles at the electrode-water interface for Cu-NR$_{DMSO}$, Cu-NR$_{IPA}$, and Cu-NR$_{EAC}$ are 146°, 141°, and 136°, respectively. The hydrophobic microenvironment formed by the –$(CF_2)_n$– backbone limits the accessibility of protons and water,[52,53] resulting in the lowest FE_{H_2} observed for Cu-NR$_{DMSO}$. Moreover, these contact angles remain essentially unchanged after 24 h of electrolysis (Figure S27), indicating that the Nafion configuration within the catalyst layer is stable under reaction conditions.

The preceding discussion elucidates that Nafion adopts diverse configurations, resulting in varied *CO adsorption behavior and HER activity. To further

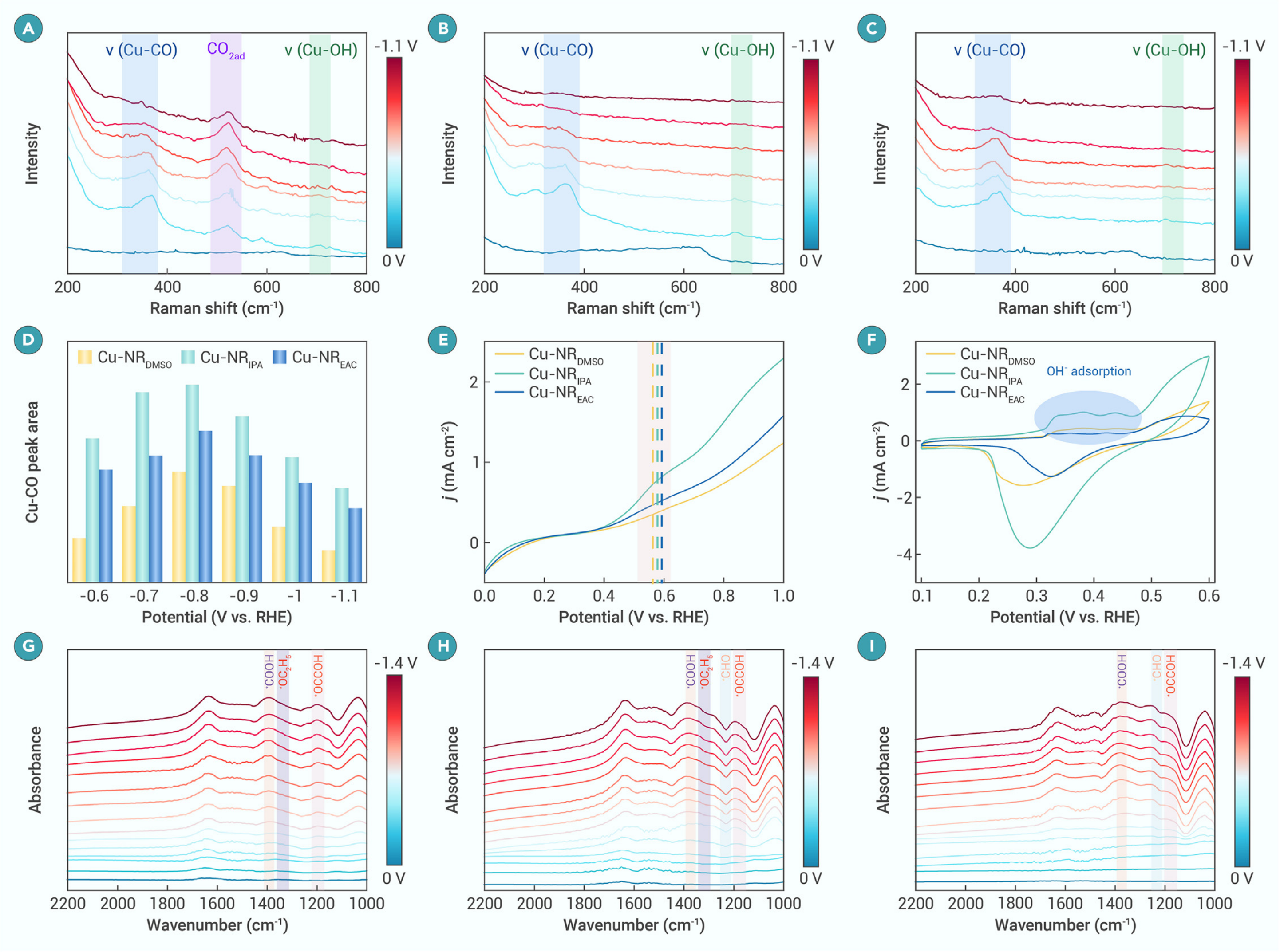

Figure 2. ***In situ*** **characterizations during CO_2RR over different electrodes** (A–C) *In situ* Raman spectra at different potentials over Cu-NR_{DMSO} (A), Cu-NR_{IPA} (B), and Cu-NR_{EAC} (C). (D) The normalized *CO peak area as a function of the applied potential over various Cu electrodes. (E)The electrochemical CO stripping curves of various Cu electrodes. (F) CV curves over various Cu electrodes in 1 M KOH. (G–I) *In situ* ATR-SEIRAS spectra at different potentials over Cu-NR_{DMSO} (G), Cu-NR_{IPA} (H), and Cu-NR_{EAC} (I).

elucidate the mechanism by which solvent choice influences specific Nafion configurations on the catalyst surface, we conducted MD simulations to examine the aggregation behavior of Nafion and its interactions with the catalyst. The radial distribution functions (RDFs), representing side chain-side chain interactions between different Nafion molecules (S-S), show pronounced differences in peak intensity across solvents with varying ε values, with peak intensity decreasing as ε increases (Figure 4A). In low-ε EAC, the peak at 2.17 Å is four times more intense than in high-ε DMSO. Similar trends are observed in RDFs representing backbone-side chain interactions between different Nafion molecules (B-S) (Figure S28), indicating that Nafion molecules aggregate more strongly in solvents with lower ε, consistent with previous reports.[54,55] This aggregation behavior is further supported by SAXS, where progressively steeper slopes are observed for Nafion in DMSO, IPA, and EAC, reflecting the formation of larger aggregates in lower-ε solvents (Figure 4B). DLS measurements reveal the hydrodynamic diameters of Nafion as 0.9, 7, and 100 nm in DMSO, IPA, and EAC, respectively (Figure 4C), confirming enhanced aggregation in low-ε solvents. Cryo-TEM images show that Nafion disperses considerably well in DMSO without forming aggregates, while it forms wormlike and spherical aggregates in IPA and EAC, respectively (Figures 4D–4F), in agreement with DLS results. The pronounced aggregation observed in low-ε solvents causes catalyst agglomeration and reduces contact angles (Figures S8 and 3F–3H). Furthermore, the ^{19}F-NMR signal of Nafion is observed in DMSO and IPA but not in EAC (Figure 4G), aligning with the DLS and SAXS data and indicating severe aggregation in EAC. In low-ε EAC, aggregation restricts the molecular rotation of Nafion, broadening the NMR signals into the baseline and obscuring fine spectral features.[56] These findings highlight that the dispersion solvent directly modulates the balance between electrostatic and hydrophobic interactions within Nafion. Higher-ε solvents enhance electrostatic repulsion, disrupting hydrophobic clustering, while lower-ε solvents allow hydrophobic interactions to dominate, leading to pronounced aggregation. Additionally, we investigated the interactions between Nafion and CuO within the catalyst ink using MD simulations. The RDF peak corresponding to CuO-backbone interactions (Cu-B) shifts to lower *r* values as ε decreases (Figure S29), indicating that the Nafion backbone increasingly adsorbs onto the CuO surface in low-ε solvents. In contrast, the RDF peak corresponding to CuO-side chain interactions (Cu-S) becomes more pronounced in high-ε solvents (Figure S30), indicating stronger side-chain adsorption onto CuO under these conditions—consistent with the Raman data shown in Figure 3A. As Nafion undergoes considerable aggregation and exhibits weaker interactions with the catalyst in low-ε solvents, the catalyst itself becomes prone to agglomeration, reducing the active surface area, consistent with SEM images (Figure S8) and C_{dl} values (Figure S17).

Nafion exhibits uniform dispersion in high-ε DMSO but forms large aggregates in low-ε EAC, resulting in particle agglomeration and a rough surface on Cu-NR_{EAC}. In DMSO, Nafion uniformly arranges on the Cu surface, with the side chains interacting directly with the Cu surface, while the $-(CF_2)_n-$ backbone extends outward, forming a hydrophobic layer (Figure 3C). Conversely, in EAC, Nafion adsorbs onto the Cu surface in the form of aggregates, with the outward-facing backbone positioned closer to the Cu surface

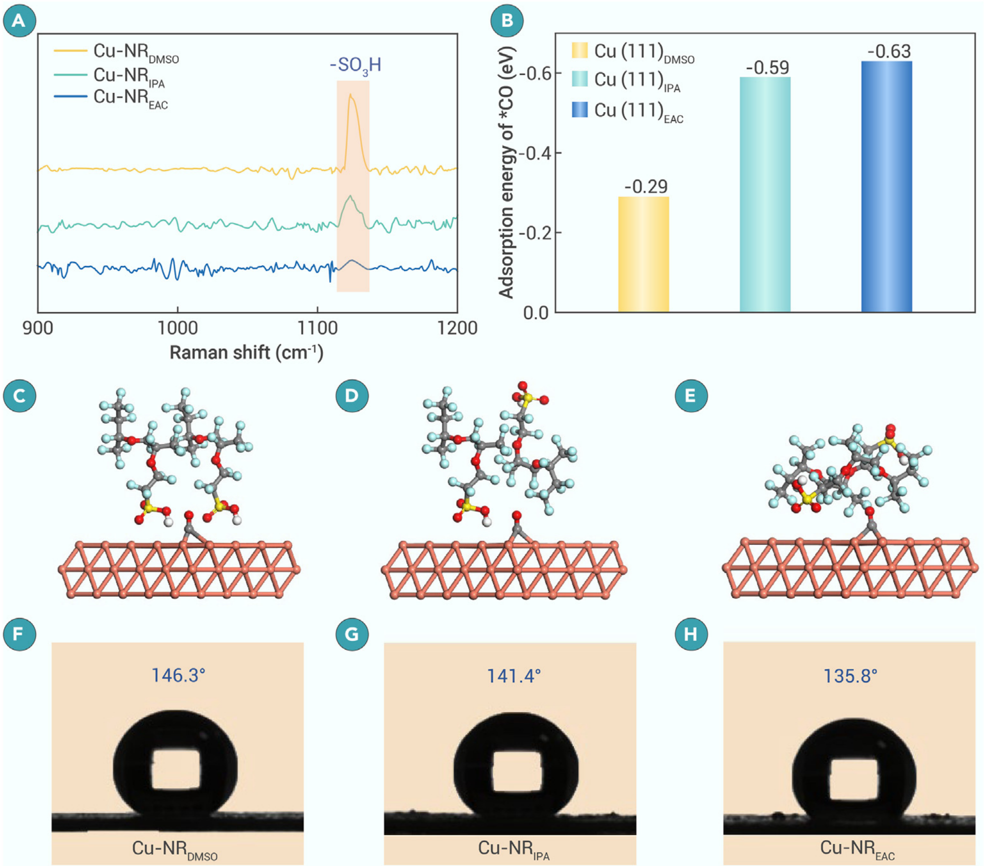

Figure 3. The configuration of Nafion molecules on the catalyst surface (A) Raman spectra of different Cu electrodes. (B) Adsorption energy of *CO intermediates on different Cu electrodes. (C–E) Binding model of CO_2 and Nafion molecules on Cu(111)$_{DMSO}$ (C), Cu(111)$_{IPA}$ (D), and Cu(111)$_{EAC}$ (E). (F–H) Contact angles between surfaces of Cu-NR electrodes and water droplets.

(Figure 3E). This configuration distances the –SO_3H groups from the surface, promoting *CO adsorption and enhancing C–C coupling and CH_4 formation. However, the aggregated –$(CF_2)_n$– chains reduce the hydrophobicity of the electrode surface, leading to an intensified HER over Cu-NR$_{EAC}$. In the moderate-ε solvent IPA, Nafion exhibits intermediate aggregation behavior (Figure 3D), resulting in balanced *CO adsorption and HER, ultimately yielding the highest FE_{C2+}. To further establish this correlation, nine commonly used solvents with varying ε values were employed to prepare Cu-NR electrodes (Table S1). The resulting CO_2RR performance reveals a clear trend: Cu-NR electrodes prepared in low-ε solvents favor both C–C coupling and the HER, with the additional capability of CH_4 production, while those prepared in high-ε solvents predominantly favor CO formation. Notably, Cu-NR electrodes prepared in moderate-ε solvents achieve the highest FE_{C2+} values (Figure S31), providing further validation of the proposed mechanism. Overall, the choice of dispersion solvent dictates the aggregation behavior of Nafion and, in turn, its configuration on the Cu surface. Both the –SO_3H group and the –$(CF_2)_n$– backbone of Nafion can inhibit *CO adsorption and the HER. In high-ε solvents, Nafion disperses very well, resulting in more isolated –SO_3H groups and –$(CF_2)_n$– backbones on the Cu surface, which enhances CO formation. Conversely, the aggregation behavior observed in low-ε solvents

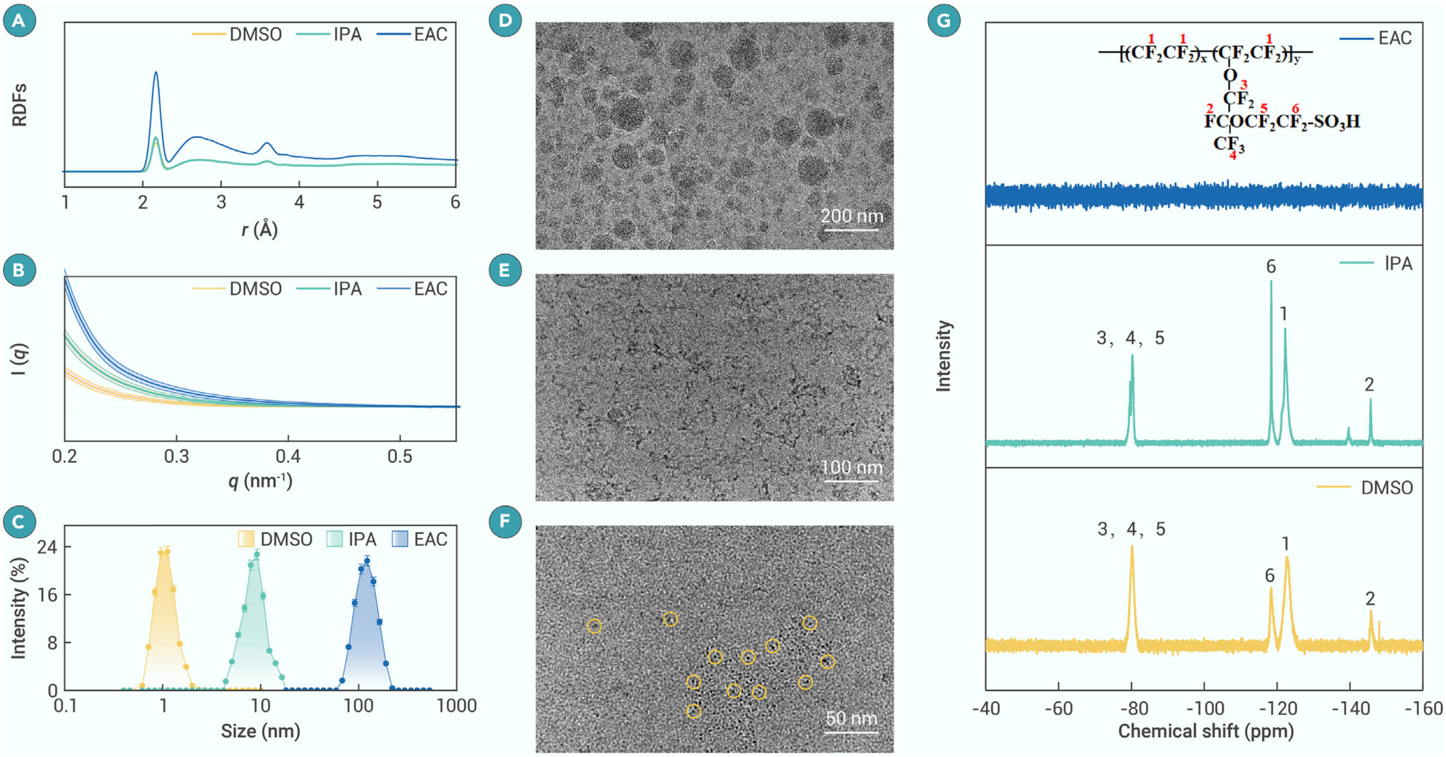

Figure 4. Aggregation behavior of Nafion molecules in different solvents (A) RDFs of S-S in various Nafion solutions. (B) SAXS curves of various Nafion solutions. (C) DLS curves of various Nafion solutions. (D–F) Cryo-TEM images of Nafion in EAC (D), IPA (E), and DMSO (F). (G) ^{19}F-NMR spectra of various Nafion solutions.

leads to fewer isolated $-SO_3H$ groups and $-(CF_2)_n-$ backbones on the Cu surface, thereby promoting both C–C coupling and the HER.

DISCUSSION

This study presents a novel approach for tailoring the surface microenvironment of electrodes for the CO_2RR by varying the solvent used to disperse the catalyst and ionomer. Nafion molecules exhibit distinct aggregation behaviors and arrangements on the Cu surface depending on the ε of the solvent. As the ε value decreases, Nafion molecules increasingly aggregate, positioning the *CO-incompatible $-SO_3H$ groups further from the Cu surface, alongside the aggregated hydrophobic $-(CF_2)_n-$ chains. This configuration enhances *CO adsorption and promotes the HER. As a result, the product distribution of the CO_2RR can be controlled simply by adjusting the dispersion solvent. This universal strategy is further validated using different Cu-based electrodes, exemplified by the Cu-NS catalyst, where FE_{C2+} increases from 67.5% to 90.5% at 800 mA cm^{-2} by changing the solvent from DMSO to IPA. We anticipate that this strategy can be broadly applied to a range of electrocatalytic reactions to manipulate ionomer arrangement on catalyst surfaces, enabling tunable product distributions.

RESOURCE AVAILABILITY

Materials availability

This study did not generate any new or unique materials or reagents. The materials generated in this study are available from the lead contact upon reasonable request.

Data and code availability

All data supporting this study are available in the manuscript or supplemental information.

FUNDING AND ACKNOWLEDGMENTS

The work was supported by the National Natural Science Foundation of China (22273108, 22293015, and 22121002), the Beijing Natural Science Foundation (2222043), the CAS Project for Young Scientists in Basic Research (YSBR-050), the ICCAS Carbon Neutral Chemistry program (CCNC-202403), and National Key Research and Development Program of China (2023YFA1507400). SAXS and XAS spectra were performed at 1W2A and 1W2B, respectively (BSRF, China).

AUTHOR CONTRIBUTIONS

Syntheses and characterizations of catalysts and electrocatalysis study, Y.Y., Z.L., and S.L.; MD calculations, J.J., M.Z., and P.Z.; collection and analysis of SXAS and XAFS data, X.T., Y.F., J.Y., H. Liu, and X.X.; mechanism analysis, J.Z., Y.X., and H. Liang; overall design and direction of the project, X.K. and B.H.; preparation of the manuscript, Y.Y., X.K., and B.H., with help from all authors.

DECLARATION OF INTERESTS

The authors declare no competing interests.

SUPPLEMENTAL INFORMATION

It can be found online at https://doi.org/10.1016/j.xinn.2025.100882.

REFERENCES

1. He, M., Sun, Y. and Han, B. (2022). Green carbon science: efficient carbon resource processing, utilization, and recycling towards carbon neutrality. *Angew. Chem. Int. Ed.* **61**:e202112835. DOI:https://doi.org/10.1002/anie.202112835.
2. Shafaat, H.S. and Yang, J.Y. (2021). Uniting Biological and Chemical Strategies for Selective CO_2 Reduction. *Nat. Catal.* **4**:928–933. DOI:https://doi.org/10.1038/s41929-021-00683-1.
3. Zhao, Y., Hao, L., Ozden, A. et al. (2023). Conversion of CO_2 to multicarbon products in strong acid by controlling the catalyst microenvironment. *Nat. Synth.* **2**:403–412. DOI:https://doi.org/10.1038/s44160-022-00234-x.
4. Yang, Y., Louisia, S., Yu, S. et al. (2023). Operando studies reveal active Cu nanograins for CO_2 electroreduction. *Nature* **614**:262–269. DOI:https://doi.org/10.1038/s41586-022-05540-0.
5. Zheng, M., Wang, P., Zhi, X. et al. (2022). Electrocatalytic CO_2-to-C_{2+} with ampere-level current on heteroatom-engineered copper via tuning *CO intermediate coverage. *J. Am. Chem. Soc.* **144**:14936–14944. DOI:https://doi.org/10.1021/jacs.2c06820.
6. Cao, X., Cha, S. and Gong, M. (2025). Interfacial electrical double layer in electrocatalytic reactions: fundamentals, characterizations and applications. *Acta Phys. Chim. Sin.* **41**:100041. DOI:https://doi.org/10.1016/j.actphy.2024.100041.
7. Liu, K., Yang, C., Wei, R. et al. (2022). Unraveling and tuning the linear correlation between CH_4 and C_2 production rates in CO_2 electroreduction. *Sci. Bull.* **67**:1042–1048. DOI:https://doi.org/10.1016/j.scib.2022.04.004.
8. Yang, P.P. and Gao, M.R. (2023). Enrichment of reactants and intermediates for electrocatalytic CO_2 reduction. *Chem. Soc. Rev.* **52**:4343–4380. DOI:https://doi.org/10.1039/d2cs00849a.
9. Zhang, J., Guo, C., Fang, S. et al. (2023). Accelerating electrochemical CO_2 reduction to multi-carbon products via asymmetric intermediate binding at confined nanointerfaces. *Nat. Commun.* **14**:1298. DOI:https://doi.org/10.1038/s41467-023-36926-x.
10. Yang, P.P., Zhang, X.L., Liu, P. et al. (2023). Highly enhanced chloride adsorption mediates efficient neutral CO_2 electroreduction over a dual-phase copper catalyst. *J. Am. Chem. Soc.* **145**:8714–8725. DOI:https://doi.org/10.1021/jacs.3c02130.
11. Rong, Y., Sang, J., Che, L. et al. (2023). Designing Electrolytes for Aqueous Electrocatalytic CO_2 Reduction. *Acta Phys. Chim. Sin.* **39**:2212027. DOI:https://doi.org/10.3866/PKU.WHXB202212027.
12. Dai, Y., Li, H., Wang, C. et al. (2023). Manipulating local coordination of copper single atom catalyst enables efficient CO_2-to-CH_4 conversion. *Nat. Commun.* **14**:3382. DOI:https://doi.org/10.1038/s41467-023-39048-6.
13. Ge, W., Chen, Y., Fan, Y. et al. (2022). Dynamically formed surfactant assembly at the electrified electrode-electrolyte interface boosting CO_2 electroreduction. *J. Am. Chem. Soc.* **144**:6613–6622. DOI:https://doi.org/10.1021/jacs.2c02486.
14. Ren, W., Xu, A., Chan, K. et al. (2022). A cation concentration gradient approach to tune the selectivity and activity of CO_2 electroreduction. *Angew. Chem. Int. Ed.* **61**:e202214173. DOI:https://doi.org/10.1002/anie.202214173.
15. Monteiro, M.C.O., Dattila, F., Hagedoorn, B. et al. (2021). Absence of CO_2 electroreduction on copper, gold and silver electrodes without metal cations in solution. *Nat. Catal.* **4**:654–662. DOI:https://doi.org/10.1038/s41929-021-00655-5.
16. Kim, C., Bui, J.C., Luo, X. et al. (2021). Tailored catalyst microenvironments for CO_2 electroreduction to multicarbon products on copper using bilayer ionomer coatings. *Nat. Energy* **6**:1026–1034. DOI:https://doi.org/10.1038/s41560-021-00960-0.
17. Su, Y., Cheng, Y., Li, Z. et al. (2024). Exploring the impact of Nafion modifier on electrocatalytic CO_2 reduction over Cu catalyst. *J. Energy Chem.* **88**:543–551. DOI:https://doi.org/10.1016/j.jechem.2023.10.010.
18. Li, L., Liu, Z., Yu, X. et al. (2023). Achieving high single-pass carbon conversion efficiencies in durable CO_2 electroreduction in strong acids via electrode structure engineering. *Angew. Chem. Int. Ed.* **62**:e202300226. DOI:https://doi.org/10.1002/anie.202300226.
19. Wang, M., Chen, H., Wang, M. et al. (2023). Tuning C_1/C_2 selectivity of CO_2 electrochemical reduction over in-situ evolved CuO/SnO_2 heterostructure. *Angew. Chem. Int. Ed.* **62**:e202306456. DOI:https://doi.org/10.1002/anie.202306456.
20. Liu, J., Li, P., Bi, J. et al. (2023). Switching between C_{2+} products and CH_4 in CO_2 electrolysis by tuning the composition and structure of rare-earth/copper catalysts. *J. Am. Chem. Soc.* **145**:23037–23047. DOI:https://doi.org/10.1021/jacs.3c05562.
21. Han, A., Yan, X., Chen, J. et al. (2022). Effects of dispersion solvents on proton conduction behavior of ultrathin Nafion films in the catalyst layers of proton exchange membrane fuel cells. *Acta Phys. Chim. Sin.* **38**:1912052. DOI:https://doi.org/10.3866/PKU.WHXB201912052.
22. Chen, F., Chen, S., Wang, A. et al. (2023). Blocking the sulfonate group in Nafion to unlock platinum's activity in membrane electrode assemblies. *Nat. Catal.* **6**:392–401. DOI:https://doi.org/10.1038/s41929-023-00949-w.
23. Welch, C., Labouriau, A., Hjelm, R. et al. (2012). Nafion in dilute solvent systems: dispersion or solution? *ACS Macro Lett.* **1**:1403–1407. DOI:https://doi.org/10.1021/mz3005204.
24. Ding, P., An, H., Zellner, P. et al. (2023). Elucidating the roles of Nafion/solvent formulations in copper-catalyzed CO_2 electrolysis. *ACS Catal.* **13**:5336–5347. DOI:https://doi.org/10.1021/acscatal.2c05235.
25. Liu, G., McLaughlin, D., Thiele, S. et al. (2023). Correlating catalyst ink design and catalyst layer fabrication with electrochemical CO_2 reduction performance. *Chem. Eng. J.* **460**:141757. DOI:https://doi.org/10.1016/j.cej.2023.141757.
26. Grimme, S. (2006). Semiempirical gga-type density functional constructed with a long-range dispersion correction. *J. Comput. Chem.* **27**:1787–1799. DOI:https://doi.org/10.1002/jcc.20495.
27. Kresse, G. and Furthmüller, J. (1996). Efficient iterative schemes for ab initio total-energy calculations using a plane-wave basis set. *Phys. Rev. B* **54**:11169–11186. DOI:https://doi.org/10.1103/PhysRevB.54.11169.
28. Blöchl, P.E. (1994). Projector augmented-wave method. *Phys. Rev. B* **50**:17953–17979. DOI:https://doi.org/10.1103/PhysRevB.50.17953.
29. Perdew, J.P. and Wang, Y. (1992). Pair-distribution function and its coupling-constant average for the spin-polarized electron gas. *Phys. Rev. B* **46**:12947–12954. DOI:https://doi.org/10.1103/PhysRevB.46.12947.
30. Monkhorst, H.J. and Pack, J.D. (1976). Special points for brillouin-zone integrations. *Phys. Rev. B* **13**:5188–5192. DOI:https://doi.org/10.1103/PhysRevB.13.5188.
31. Grimme, S., Antony, J., Ehrlich, S. et al. (2010). A consistent and accurate ab initio parametrization of density functional dispersion correction (DFT-D) for the 94 elements H-Pu. *J. Chem. Phys.* **132**:154104. DOI:https://doi.org/10.1063/1.3382344.
32. Grimme, S., Ehrlich, S. and Goerigk, L. (2011). Effect of the damping function in dispersion corrected density functional theory. *J. Comput. Chem.* **32**:1456–1465. DOI:https://doi.org/10.1002/jcc.21759.
33. Hess, B., Kutzner, C., van der Spoel, D. et al. (2008). GROMACS 4: Algorithms for highly efficient, load-balanced, and scalable molecular simulation. *J. Chem. Theor. Comput.* **4**:435–447. DOI:https://doi.org/10.1021/ct700301q.
34. Zhang, Q., Dong, S., Shao, P. et al. (2022). Covalent organic framework-based porous ionomers for high-performance fuel cells. *Science* **378**:181–186. DOI:https://doi.org/10.1126/science.abm6304.

35. Florence, A.T., Arunothayanun, P., Kiri, S. et al. (1999). Some rheological properties of nonionic surfactant vesicles and the determination of surface hydration. *J. Phys. Chem. B* **103**:1995–2000. DOI:https://doi.org/10.1021/jp983110p.
36. Malek, K., Eikerling, M., Wang, Q. et al. (2007). Self-organization in catalyst layers of polymer electrolyte fuel cells. *J. Phys. Chem. C* **111**:13627–13634. DOI:https://doi.org/10.1021/jp072692k.
37. Bussi, G., Donadio, D. and Parrinello, M. (2007). Canonical sampling through velocity rescaling. *J. Chem. Phys.* **126**:014101. DOI:https://doi.org/10.1063/1.2408420.
38. Schalenbach, M., Hoefner, T., Paciok, P. et al. (2015). Gas permeation through Nafion. Part 1: Measurements. *J. Phys. Chem. C* **119**:25145–25155. DOI:https://doi.org/10.1021/acs.jpcc.5b04155.
39. Yin, Y., Peng, Y., Zhou, M. et al. (2023). Highly efficient zinc electrode prepared by electrodeposition in a salt-induced pre-phase separation region solution. *Sci. Bull.* **68**:2362–2369. DOI:https://doi.org/10.1016/j.scib.2023.08.042.
40. Wu, M., Huang, D., Lai, F. et al. (2023). Sequential *CO management via controlling in situ reconstruction for efficient industrial-current-density CO_2-to-C_{2+} electroreduction. *Proc. Natl. Acad. Sci. USA* **120**:e2302851120. DOI:https://doi.org/10.1073/pnas.2302851120.
41. Jia, S., Dong, M., Zhu, Q. et al. (2024). Electrochemical conversion of CO_2 via C—X bond formation: recent progress and perspective. *Chem. Synth.* **4**:60. DOI:https://doi.org/10.20517/cs.2024.32.
42. He, M., Chang, X., Chao, T.H. et al. (2022). Selective Enhancement of methane formation in electrochemical CO_2 reduction enabled by a Raman-inactive oxygen-containing species on Cu. *ACS Catal.* **12**:6036–6046. DOI:https://doi.org/10.1021/acscatal.2c00087.
43. Cao, Y., Chen, Z., Li, P. et al. (2023). Surface hydroxide promotes CO_2 electrolysis to ethylene in acidic conditions. *Nat. Commun.* **14**:2387. DOI:https://doi.org/10.1038/s41467-023-37898-8.
44. Li, P., Bi, J., Liu, J. et al. (2023). p-d orbital hybridization induced by p-block metal-doped Cu promotes the formation of C_{2+} products in ampere-level CO_2 electroreduction. *J. Am. Chem. Soc.* **145**:4675–4682. DOI:https://doi.org/10.1021/jacs.2c12743.
45. Soodi, S., Zhang, J.J., Zhang, J. et al. (2024). Selective electroreduction of CO_2 to C_{2+} products on cobalt decorated copper catalysts. *Chem. Synth.* **4**:44. DOI:https://doi.org/10.20517/cs.2024.11.
46. Amirbeigiarab, R., Tian, J., Herzog, A. et al. (2023). Atomic-scale surface restructuring of copper electrodes under CO_2 electroreduction conditions. *Nat. Catal.* **6**:837–846. DOI:https://doi.org/10.1038/s41929-023-01009-z.
47. Bai, H., Cheng, T., Li, S. et al. (2021). Controllable CO adsorption determines ethylene and methane productions from CO_2 electroreduction. *Sci. Bull.* **66**:62–68. DOI:https://doi.org/10.1016/j.scib.2020.06.023.
48. Zhou, Y., Martín, A.J., Dattila, F. et al. (2022). Long-chain hydrocarbons by CO_2 electroreduction using polarized nickel catalysts. *Nat. Catal.* **5**:545–554. DOI:https://doi.org/10.1038/s41929-022-00803-5.
49. Han, Z., Han, D., Chen, Z. et al. (2022). Steering surface reconstruction of copper with electrolyte additives for CO_2 electroreduction. *Nat. Commun.* **13**:3158. DOI:https://doi.org/10.1038/s41467-022-30819-1.
50. Xie, Y., Ou, P., Wang, X. et al. (2022). High carbon utilization in CO_2 reduction to multi-carbon products in acidic media. *Nat. Catal.* **5**:564–570. DOI:https://doi.org/10.1038/s41929-022-00788-1.
51. Ott, S., Orfanidi, A., Schmies, H. et al. (2020). Ionomer distribution control in porous carbon-supported catalyst layers for high-power and low Pt-loaded proton exchange membrane fuel cells. *Nat. Mater.* **19**:77–85. DOI:https://doi.org/10.1038/s41563-019-0487-0.
52. Li, Y., Pei, Z., Luan, D. et al. (2023). Superhydrophobic and conductive wire membrane for enhanced CO_2 electroreduction to multicarbon products. *Angew. Chem. Int. Ed.* **62**:e202302128. DOI:https://doi.org/10.1002/anie.202302128.
53. Sheng, X., Ge, W., Jiang, H. et al. (2022). Engineering the Ni-N-C catalyst microenvironment enabling CO_2 electroreduction with nearly 100% CO selectivity in acid. *Adv. Mater.* **34**:2201295. DOI:https://doi.org/10.1002/adma.202201295.
54. Lee, J.H., Doo, G., Kwon, S.H. et al. (2018). Dispersion-solvent control of ionomer aggregation in a polymer electrolyte membrane fuel cell. *Sci. Rep.* **8**:10739. DOI:https://doi.org/10.1038/s41598-018-28779-y.
55. Mabuchi, T., Huang, S.F. and Tokumasu, T. (2020). Dispersion of Nafion ionomer aggregates in 1-propanol/water solutions: Effects of ionomer concentration, alcohol content, and salt addition. *Macromolecules* **53**:3273–3283. DOI:https://doi.org/10.1021/acs.macromol.9b02725.
56. Kim, T.H., Yi, J.Y., Jung, C.Y. et al. (2017). Solvent effect on the Nafion agglomerate morphology in the catalyst layer of the proton exchange membrane fuel cells. *Int. J. Hydrogen. Energy.* **42**:478–485. DOI:https://doi.org/10.1016/j.ijhydene.2016.12.015.

The Innovation, Volume *6*

Supplemental Information

Modulating CO_2 electroreduction pathways through controlled ionomer arrangement on catalyst surfaces via solvent dispersion

Yaoyu Yin, Zhongnan Ling, Shiqiang Liu, Jiapeng Jiao, Meng Zhou, Pei Zhang, Xing Tong, Yueqian Fan, Jiahao Yang, Huanyan Liu, Xueqing Xing, Jianling Zhang, Yi Xu, Hongyan Liang, Xinchen Kang, and Buxing Han

Supplementary Figures

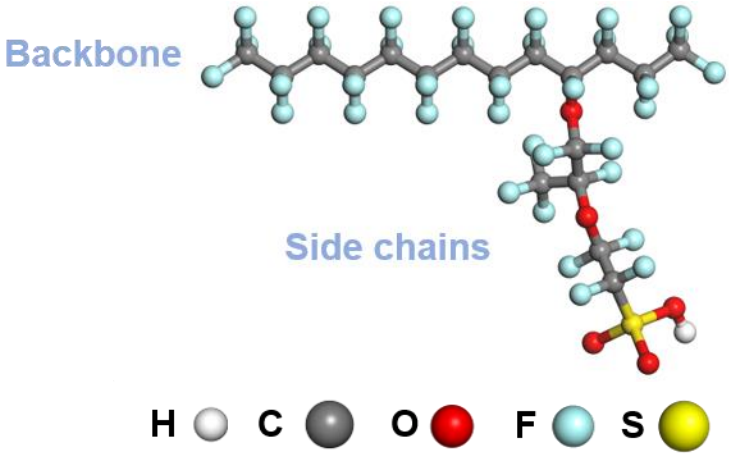

Figure S1. The structure of Nafion monomer..

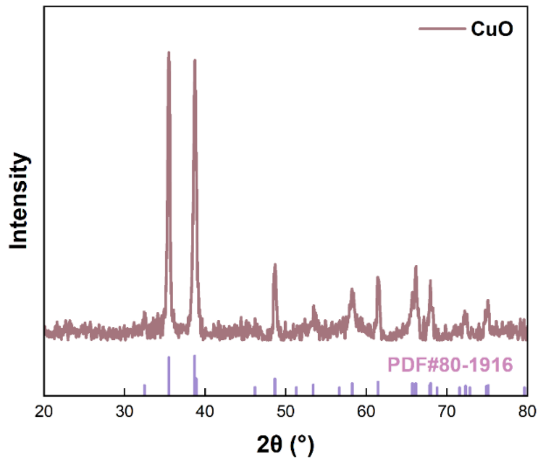

Figure S2. XRD pattern of CuO nanorods.

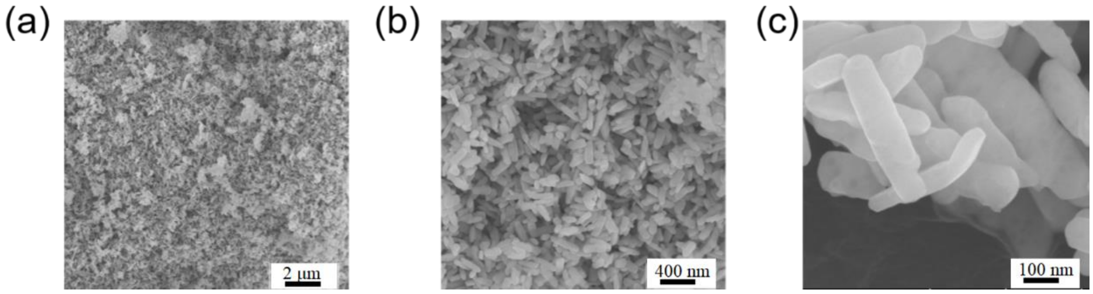

Figure S3. SEM images of CuO nanorods.

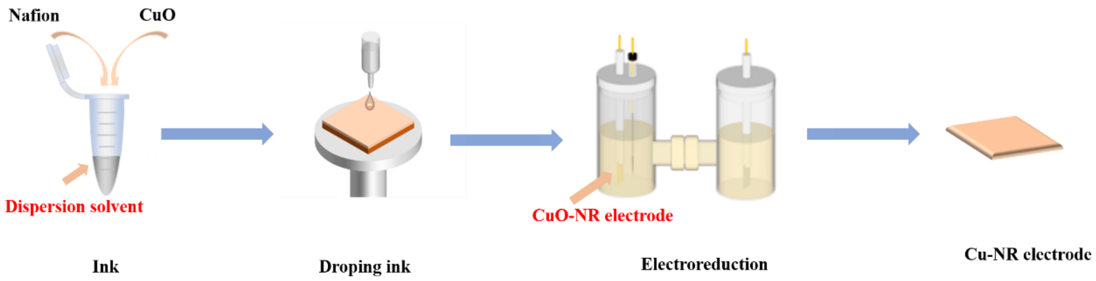

Figure S4. Schematic diagram for the preparation of Cu-NR electrodes.

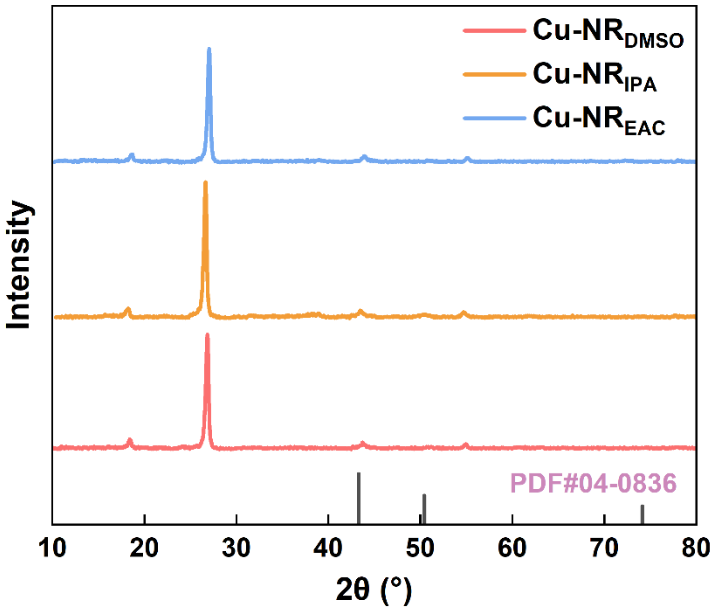

Figure S5. XRD pattern of Cu-NR$_{DMSO}$ (a), Cu-NR$_{IPA}$ (b) and Cu-NR$_{EAC}$ (c).

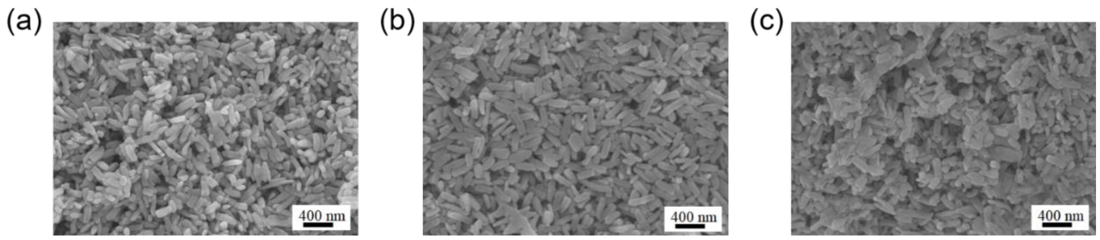

Figure S6. SEM images of Cu-NR$_{DMSO}$ (a), Cu-NR$_{IPA}$ (b) and Cu-NR$_{EAC}$ (c).

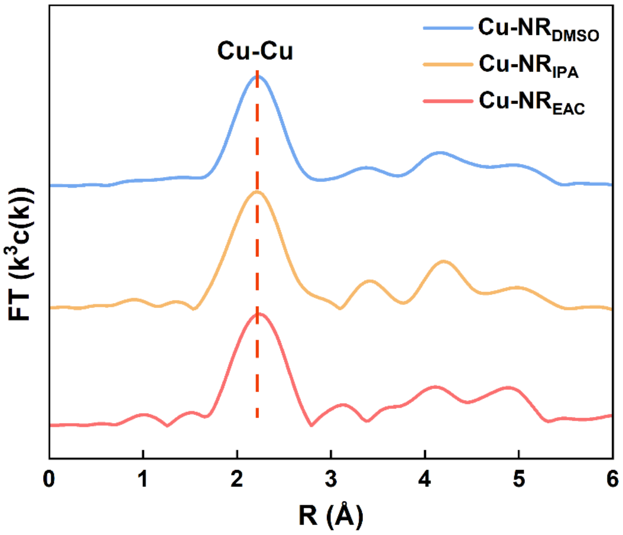

Figure S7. The Cu K-edge XANES spectra of different Cu electrodes.

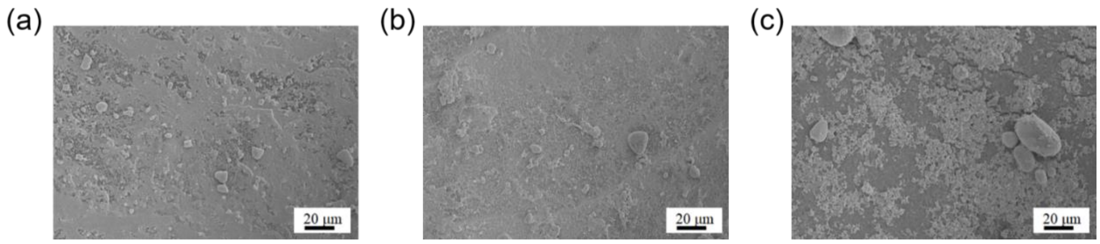

Figure S8. SEM images of Cu-NR$_{DMSO}$ (a–b), Cu-NR$_{IPA}$ (c–d) and Cu-NR$_{EAC}$ (e–f).

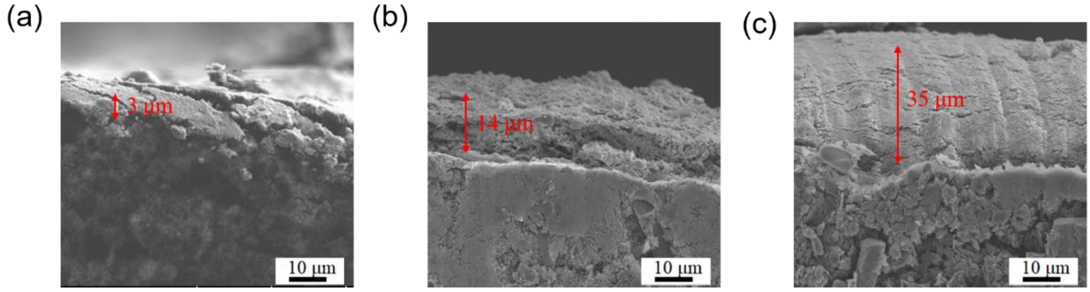

Figure S9. SEM images from side view of Cu-NR$_{DMSO}$ (a), Cu-NR$_{IPA}$ (b) and Cu-NR$_{EAC}$ (c).

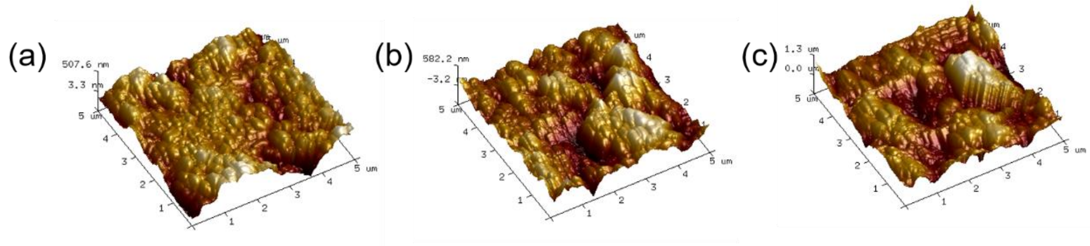

Figure S10. 3D AFM images of Cu-NR$_{DMSO}$ (a), Cu-NR$_{IPA}$ (b) and Cu-NR$_{EAC}$ (c).

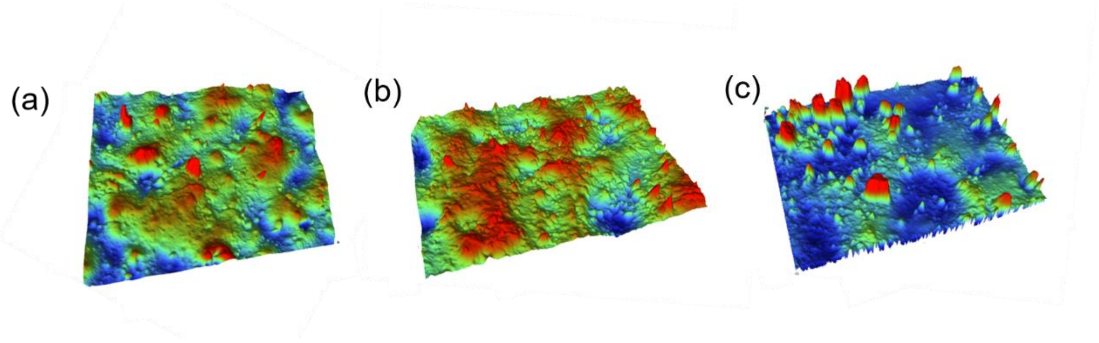

Figure S11. 3D Profiling images of Cu-NR$_{DMSO}$ (a), Cu-NR$_{IPA}$ (b) and Cu-NR$_{EAC}$ (c).

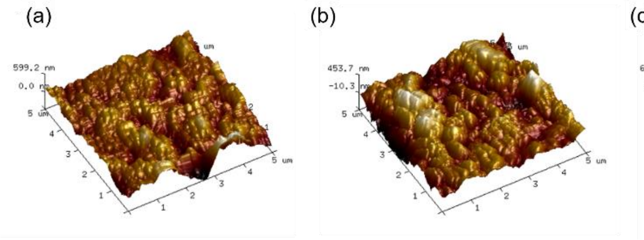

Figure S12. 3D AFM images of CuO-NR$_{DMSO}$ (a), CuO-NR$_{IPA}$ (b) and CuO-NR$_{EAC}$ (c).

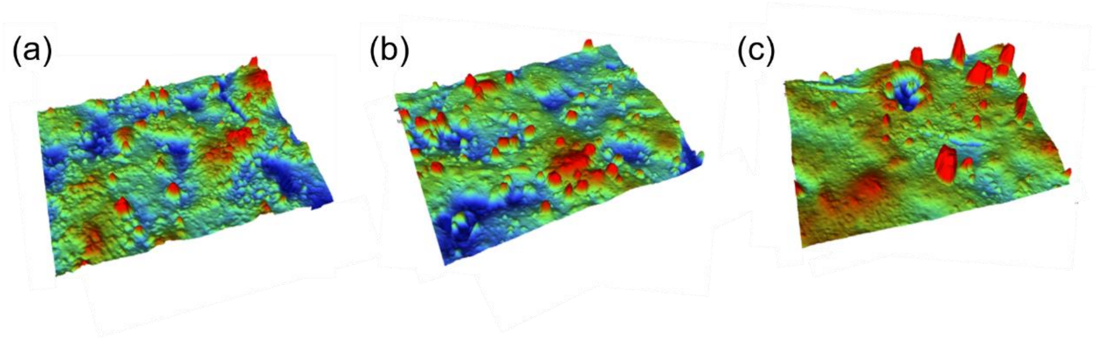

Figure S13. 3D Profiling images of CuO-NR$_{DMSO}$ (a), CuO-NR$_{IPA}$ (b) and CuO-NR$_{EAC}$ (c).

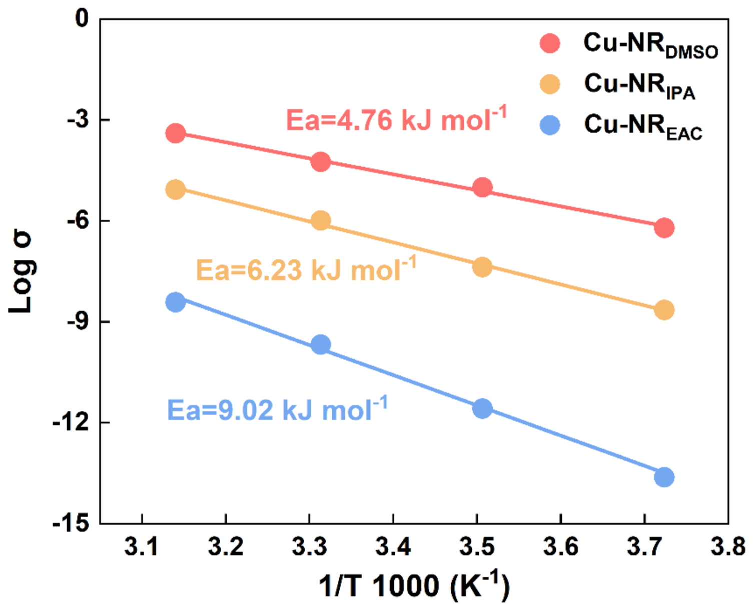

Figure S14. Relationship between conductivity and temperature.

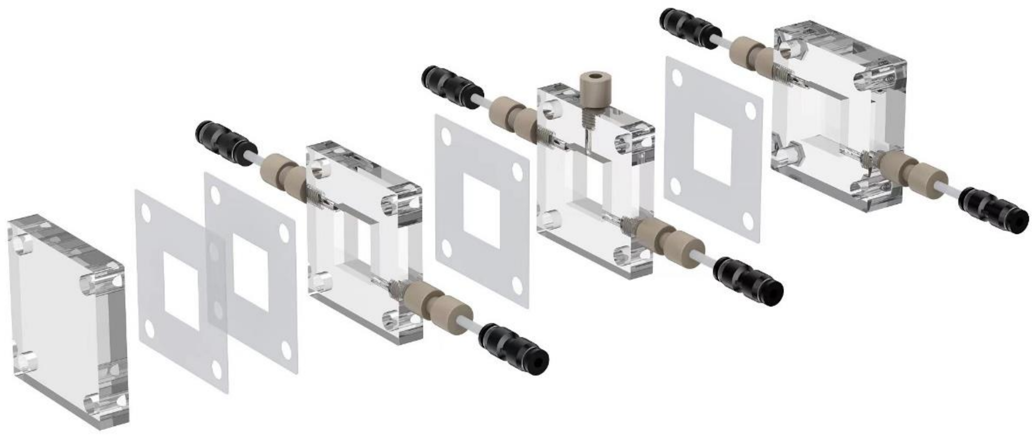

Figure S15. Schematic diagram of the flow cell used for CO_2RR.

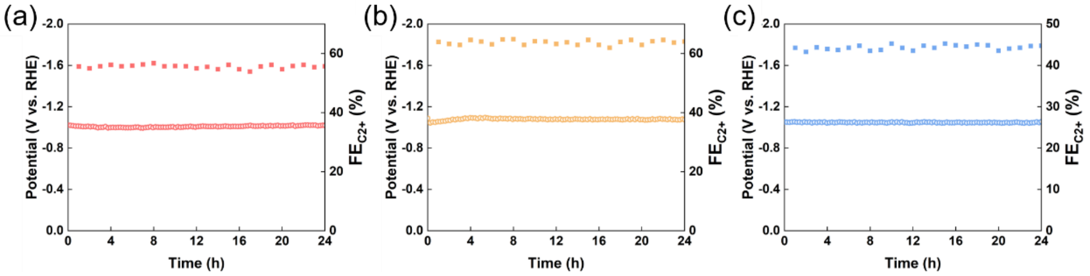

Figure S16. Plot of potential (hollow circle) and FE_{C2+} (solid circle) vs. time over Cu-NR_{DMSO} (a), Cu-NR_{IPA} (b) and Cu-NR_{EAC} (c) at 800 mA cm^{-2}.

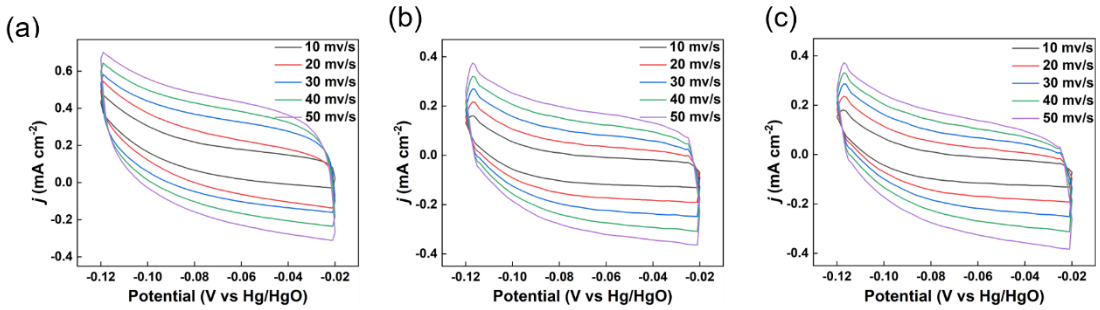

Figure S17. CV scans at the non-Faradaic region with different scan rates over Cu-NR_{DMSO} (a), Cu-NR_{IPA} (b) and Cu-NR_{EAC} (c).

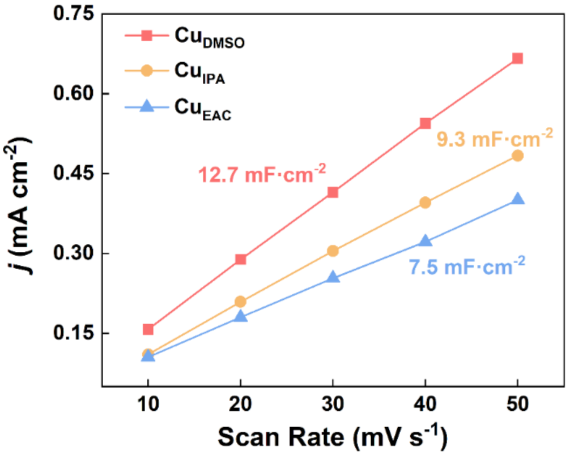

Figure S18. Plot of difference in current density vs. scan rate.

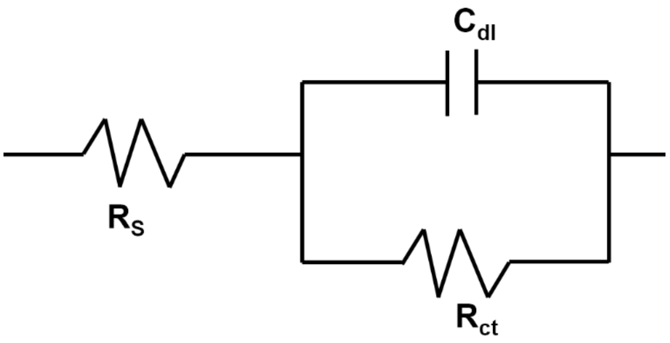

Figure S19. Electrical equivalent circuit used for simulating the EIS data.

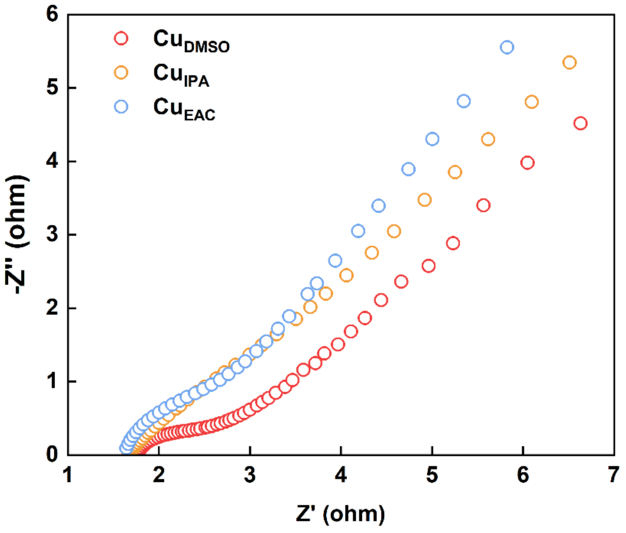

Figure S20. Nyquist plots using different electrodes.

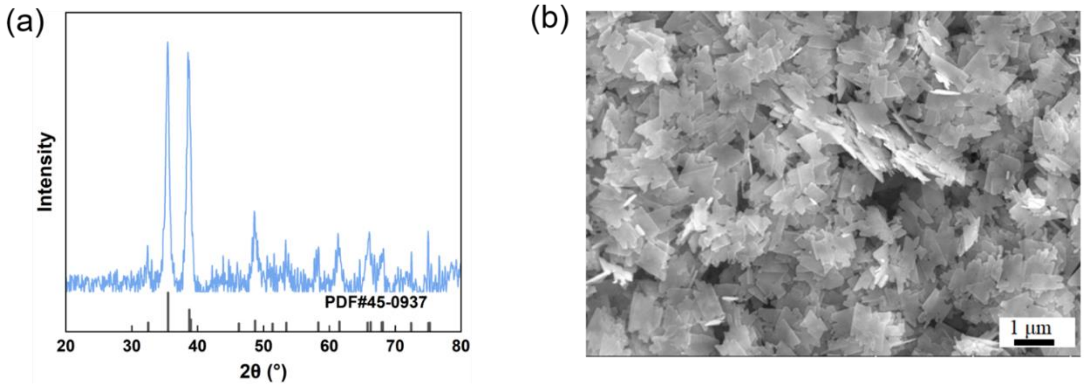

Figure S21. XRD pattern (a) and SEM image (b) of CuO-NS.

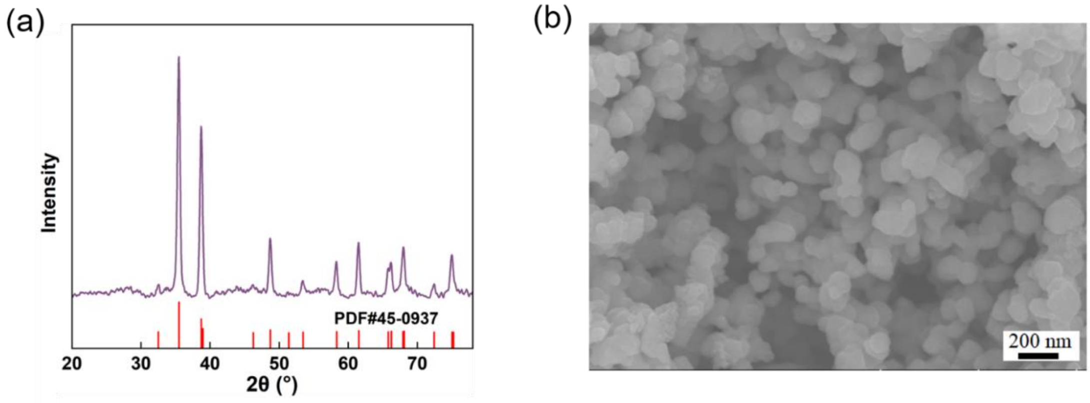

Figure S22. XRD pattern (a) and SEM image (b) of CuO-NP.

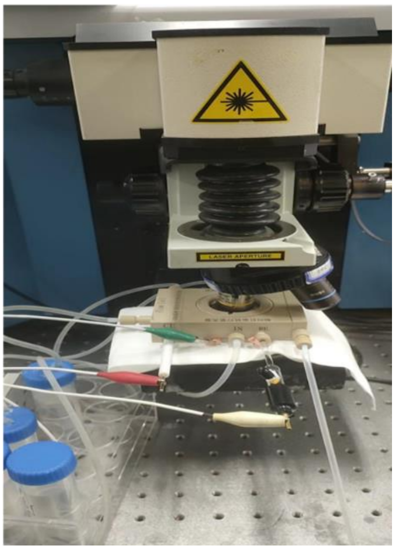

Figure S23. The in situ electrochemical spectral cell for Raman test.

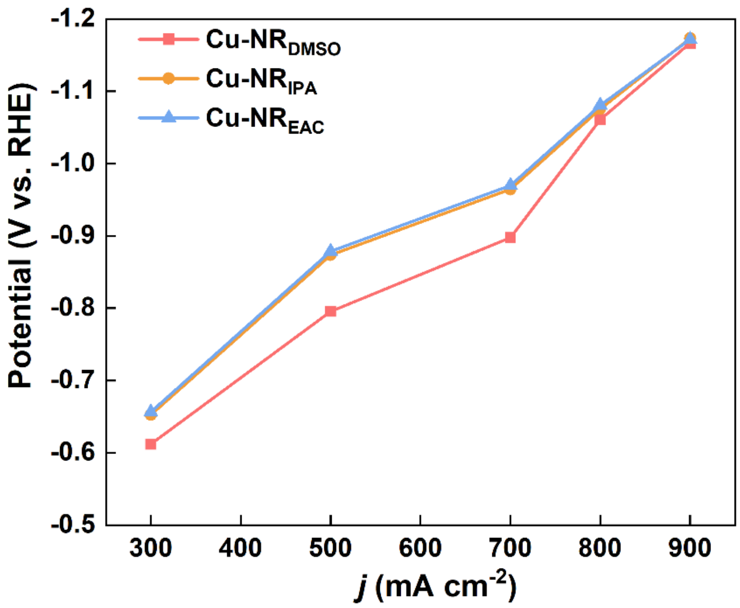

Figure S24. Plot of potential vs. current density over different electrodes.

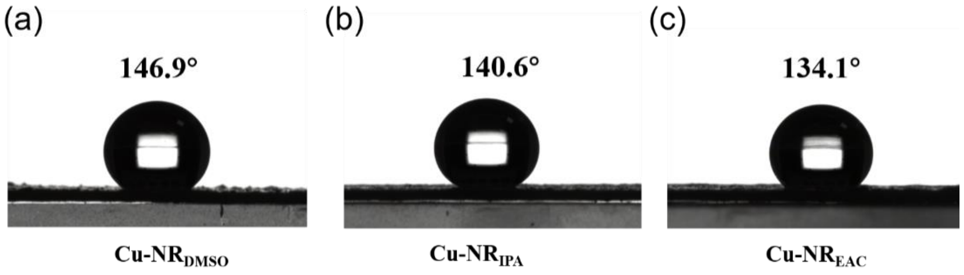

Figure S25. Contact angles between surfaces of CuO-NR electrodes and water droplet.

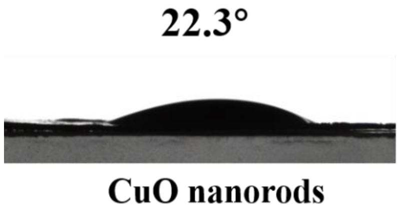

Figure S26. Contact angles between surfaces of CuO-NR and water droplet.

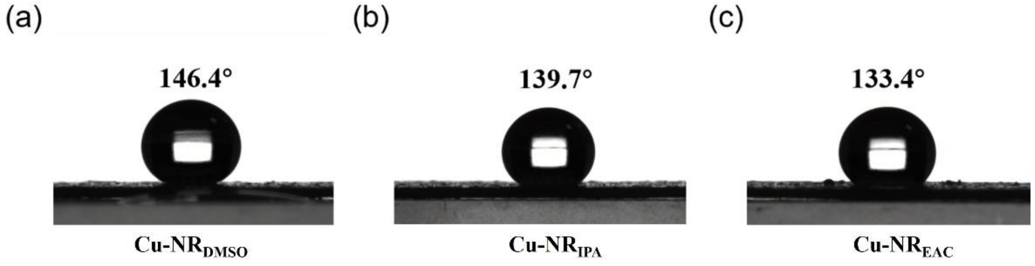

Figure S27. Contact angles between surfaces of Cu-NR electrodes and water droplet after 24 hours of electrolysis.

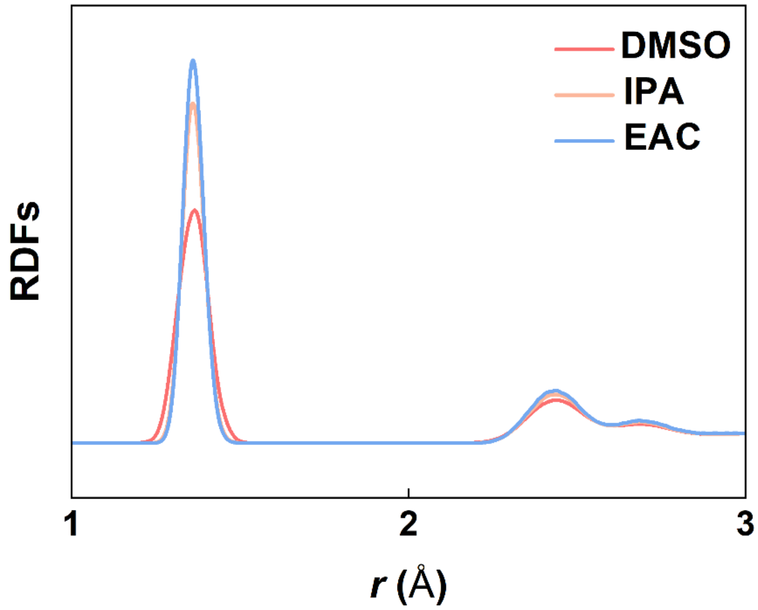

Figure S28. RDFs of B–S in different solvents.

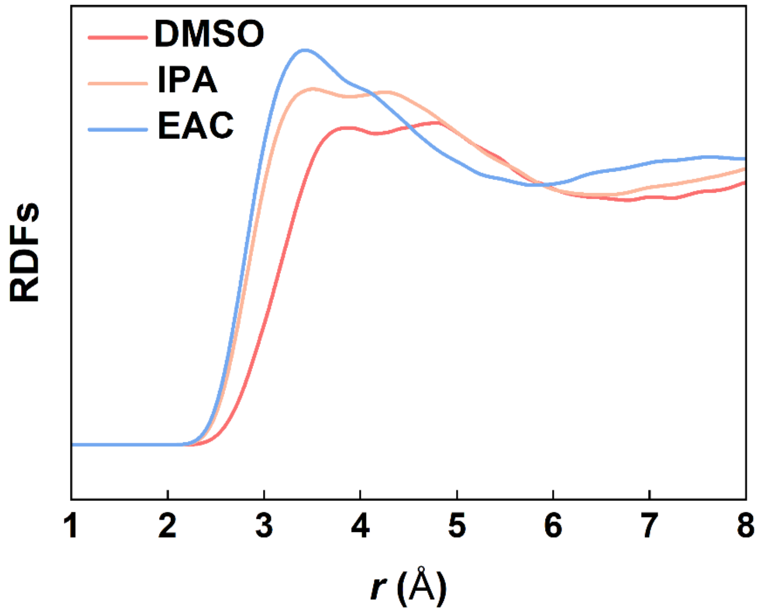

Figure S29. RDFs of Cu–B in different solvents.

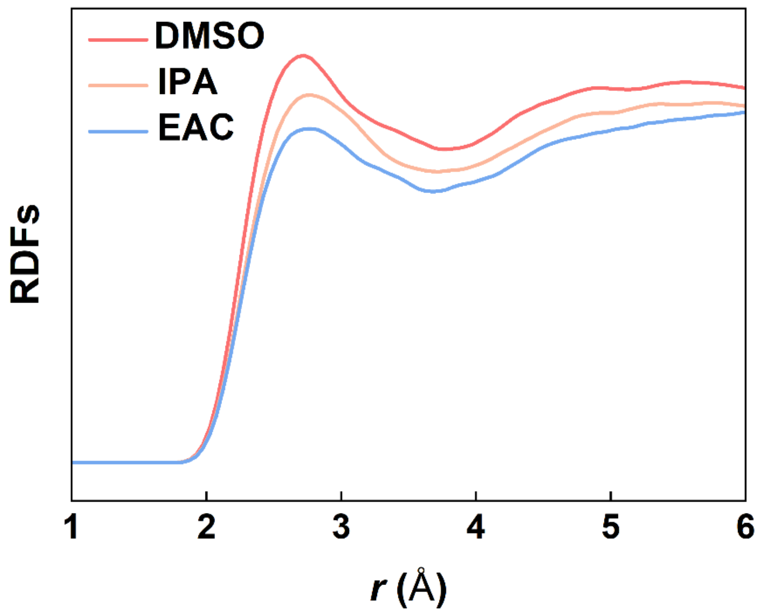

Figure S30. RDFs of Cu–S in different solvents.

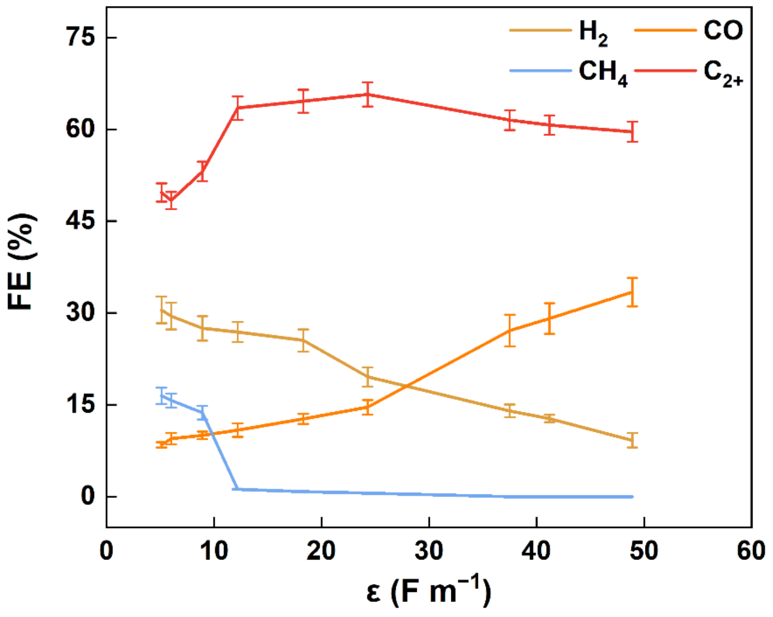

Figure S31. FEs of different products over various Cu-NR electrodes prepared in solvents with varying ε values at 800 mA cm^{-2}.

Supplementary Tables

Table S1. The abbreviation and ε values of different solutions.

Solutions	Abbreviation	ε (F m^{-1})
Chloroform	TCM	5.1
Ethyl acetate	EAC	6.02
Dichloromethane	MC	8.9
Butanol	TBA	12.5
2-propanol	IPA	18.3
Ethanol	EA	24.3
Ethylene glycol	EG	37.7
2-furaldehyde	FD	41.9
Dimethyl sulfoxide	DMSO	48.9
